# PHOSPHO1 Suppresses Ferroptosis in Retinal Pigment Epithelial Cells by Reducing the Levels of Phosphatidylethanolamine Molecular Species

**DOI:** 10.1002/advs.202505359

**Published:** 2025-05-21

**Authors:** Zhiyang Chen, Xiaoman Zhu, Michael Mingze Lu, Qingjian Ou, Xueying Wang, Zhenzhen Zhao, Qi Shen, Qian Wang, Zhe Wang, Jing‐Ying Xu, Caixia Jin, Furong Gao, Juan Wang, Jingfa Zhang, Jieping Zhang, Xiaoliang Jin, Yanlong Bi, Lixia Lu, Guo‐Tong Xu, Haibin Tian

**Affiliations:** ^1^ Department of Ophthalmology of Tongji Hospital and Laboratory of Clinical and Visual Sciences of Tongji Eye Institute School of Medicine Tongji University Shanghai 200065 China; ^2^ Department of Physiology and Pharmacology School of Medicine Tongji University Shanghai 200092 China; ^3^ Department of Physiology College of Basic Medical Sciences Naval Medical University Shanghai 200433 China; ^4^ The International Eye Research Institute of the Chinese University of Hong Kong (Shenzhen) Shenzhen 518000 China; ^5^ Department of Ophthalmology Ninth People's Hospital, Shanghai Jiao Tong University School of Medicine Shanghai 200025 China

**Keywords:** age‐related macular degeneration, ferroptosis, phosphatidylethanolamine, phosphoethanolamine/phosphocholine phosphatase 1, retinal pigment epithelial cells

## Abstract

Iron‐induced lipid peroxidation of phosphatidylethanolamine (PE) species is a key driver of ferroptosis in retinal pigment epithelial (RPE) cells, a process closely associated with age‐related macular degeneration (AMD). The previous studies have demonstrated that induced retinal pigment epithelial (iRPE) cells generated by transcription factor‐mediated reprogramming exhibit superior therapeutic efficacy in treating AMD. In this study, it is found that these iRPE cells are resistant to ferroptosis and further identified phosphoethanolamine/phosphocholine phosphatase 1 (PHOSPHO1) as a critical regulator underlying ferroptosis resistance. Mechanistically, PHOSPHO1 inhibits ferroptosis through two distinct mechanisms. First, it reduces PE levels in the endoplasmic reticulum, thereby limiting PE‐derived lipid peroxidation. Second, it suppresses autophagy and ferritinophagy, leading to a reduction in intracellular free iron accumulation. Experiments using an in vivo rat model confirm that PHOSPHO1 effectively protects RPE cells from ferroptotic damage. These findings highlight PHOSPHO1 as a potential therapeutic target for AMD, providing insights into novel ferroptosis‐based intervention strategies.

## Graphical abstract

Iron‐induced lipid peroxidation of phosphatidylethanolamine (PE) species is the key driver of ferroptosis. The authors discovered that PHOSPHO1 lowers the level of PE in retinal pigment epithelial cells thus reducing the formation of lipid peroxides. Lowering PE levels can also prevent the creation of autolysosomes, reduce the production of free iron, and consequently decrease the amount of lipid peroxides.

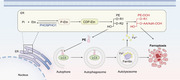

## 1 Introduction

Ferroptosis of retinal pigment epithelial cells (RPE) is one of the primary etiologies of age‐related macular degeneration (AMD).^[^ ^1^, ^2^, ^3^, ^4^ ^]^ The accumulation of iron ions that leads to lipid peroxidation is thought to cause ferroptosis.^[^ ^5^ ^]^ There are two main processes for producing lipid peroxides: a non‐enzymatic process assisted by the Fenton reaction of iron ions, and a process catalyzed by iron‐containing lipoxygenases (LOX).^[^ ^5^ ^]^ Therefore, strategies such as reducing free iron levels, blocking LOXs, or eliminating produced lipid peroxides have been confirmed to be effective at preventing ferroptosis.^[^ ^6^, ^7^, ^8^, ^9^, ^10^, ^11^, ^12^ ^]^

Previous studies have demonstrated that phosphatidylethanolamine (PE) molecules carrying polyunsaturated fatty acid side chains, arachidonic acid (AA) or adrenic acid (AdA), in the endoplasmic reticulum (ER) are the main source of the lipid peroxides that cause ferroptosis.^[^ ^13^, ^14^ ^]^ Additionally, ferroptosis has been shown to be promoted by autophagy,^[^ ^15^, ^16^ ^]^ where a crucial stage is the coupling of microtubule‐associated protein 1A/1B‐light chain 3‐I (LC3‐I) and PE to create the microtubule‐associated protein 1A/1B‐light chain 3‐II (LC3‐II) needed in the formation of the autophagosomes,^[^ ^17^ ^]^ and further studies demonstrated that autophagy can promote ferroptosis.^[^ ^15^, ^16^ ^]^ Therefore, in theory, lowering PE levels can reduce lipid peroxide formation while suppressing autophagy, thereby inhibiting ferroptosis in RPE cells.

We previously transformed dedifferentiated induced pluripotent stem cell‐derived RPE (De‐iPSC‐RPE) cells into induced RPE (iRPE) cells using key transcription factors. The iRPE cells demonstrated anti‐epithelial‐to‐mesenchymal‐transition (EMT) function and displayed better therapeutic functions.^[^ ^18^ ^]^ Considering that the cells in mesenchymal state are more susceptible to ferroptosis,^[^ ^19^ ^]^ it is very likely that iRPE cells possess enhanced resistance to ferroptosis that entails better therapeutic functions. In this study, we discovered that iRPE cells demonstrate lower PE levels in the ER and display heightened resistance to ferroptosis compared with De‐iPSC‐RPE and induced pluripotent stem cell‐derived RPE (iPSC‐RPE) cells. Further investigation revealed high expression of phosphoethanolamine/phosphocholine phosphatase 1 (PHOSPHO1) in iRPE cells, which inhibits ferroptosis in RPE cells by reducing PE levels. Therefore, our findings indicate that moderately decreasing PE levels in RPE cells can prevent ferroptosis, offering a new strategy for treating AMD.

## 2 Results

### 2.1 iRPE Cells are More Resistant to Ferroptosis than De‐iPSC‐RPE Cells and iPSC‐RPE Cells

**Figure 1:**
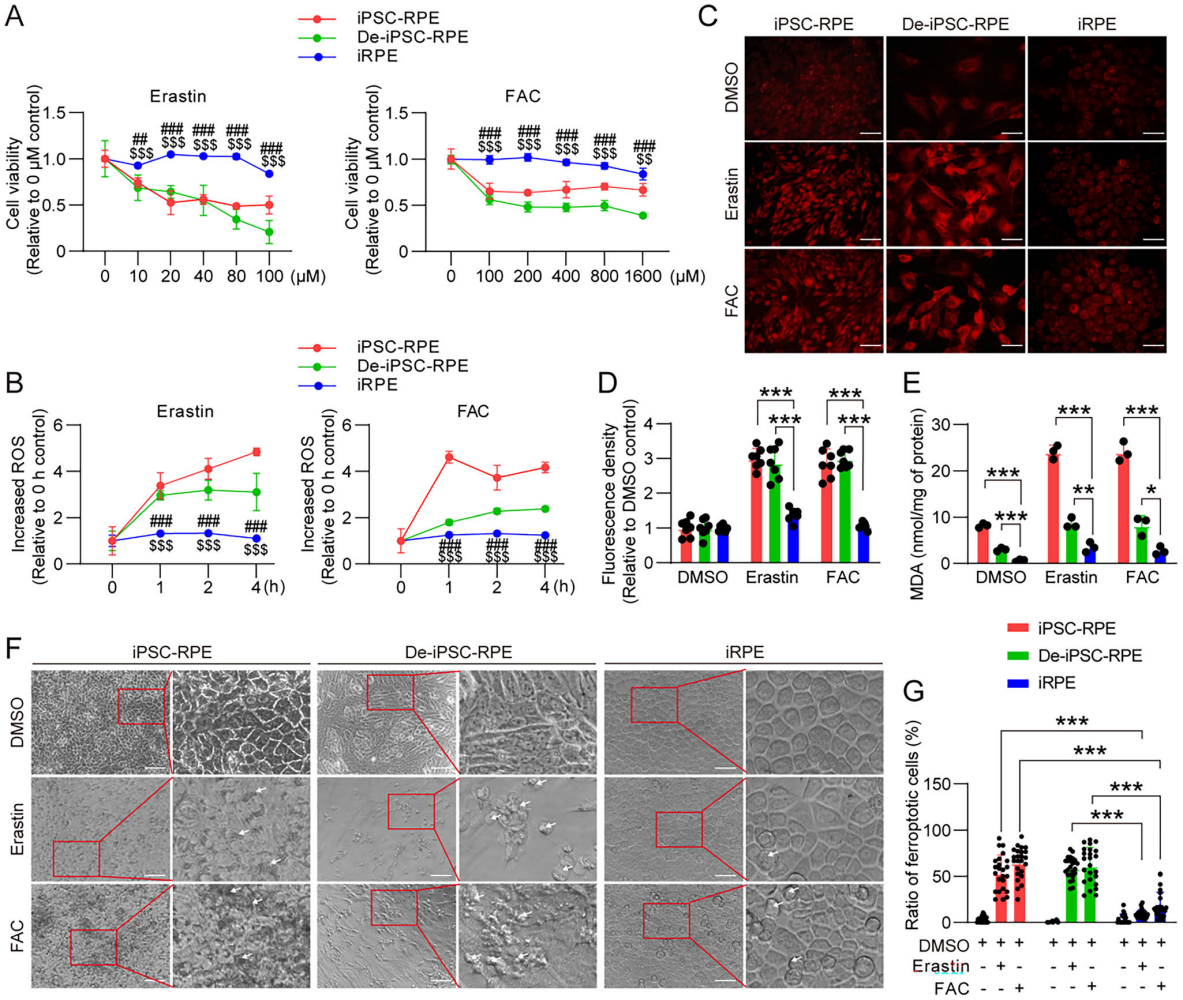
iRPE cells are more resistant to ferroptosis than De‐iPSC‐RPE cells and iPSC‐RPE cells. A) Ferroptosis was induced by erastin or FAC with different doses (*n* = 6 per dose), and cell viability was analyzed by the CCK‐8 after cells were treated for 2 days. B) Intracellular ROS was assessed using the DCFH‐DA probe after being treated with 30 µM erastin (*n* = 6) or 500 µM FAC for 4 h (*n* = 5). C,D) Intracellular Fe^2+^ levels under 30 µM erastin or 500 µM FAC treatment for 4 h were detected with (C) the RhoNox‐1 fluorescent probe and D) quantified as fluorescence density relative to the DMSO control (*n* = 7). Scale bar = 50 µm. E) The MDA in cells treated with 30 µM erastin or 500 µ FAC for 4 h was measured by the MDA detection kit and determined as nmol/mg of protein (*n* = 3). F) Representative images of ferroptotic cells among iPSC‐RPE cells, De‐iPSC‐RPE cells, and iRPE cells treated with 30 µ erastin or 500 µ FAC for 2 days. G) The ratio of ferroptotic cells was quantified as the number of dead cells over the total number of cells per field (*n* = 24). Arrows pointed the ferroptotic cells. Scale bar = 50µm. Data are mean ± SD, **P < 0.05, **P < 0.01, ***P < 0.001; $$*P* < 0.01, $$$*P* < 0.001 compared with iPSC‐RPE group; ##*P* < 0.01, ###*P* < 0.001 compared with De‐iPSC‐RPE group using one‐way ANOVA and post hoc Bonferroni's test. CCK‐8: Cell Counting Kit‐8, FAC: ferric ammonium citrate.

We previously transformed De‐iPSC‐RPE cells into iRPE cells that could maintain epithelial phenotype with EMT resistance.^[^ ^18^ ^]^ Considering that cells in the epithelial state are more resistant to ferroptosis,^[^ ^19^ ^]^ we used erastin to induce ferroptosis, aiming to determine whether iRPE cells are more resistant to ferroptosis than De‐iPSC‐RPE cells. We found that the viability of De‐iPSC‐RPE cells was significantly decreased at 10 µm erastin, whereas the viability of iRPE cells did not begin to decrease until 100 µM erastin (**Figure** **1**A). This suggests that iRPE cells are more resistant to erastin‐induced ferroptosis. Additionally, we observed a subdued increase in ferroptosis‐related molecules, including Reactive Oxygen Species (ROS) (Figure 1B), free iron ions (Figure 1C,D), and lipid peroxide intermediate malondialdehyde (MDA) (Figure 1E), in iRPE cells compared with De‐iPSC‐RPE cells. Subsequent use of ferric ammonium citrate (FAC) to trigger ferroptosis in the cells^[^ ^20^ ^]^ reinforced the finding that iRPE cells demonstrate increased ferroptosis resistance compared with De‐iPSC‐RPE cells (Figure 1A–E). We also found that iRPE cells were more resistant to ferroptosis than iPSC‐RPE cells (Figure 1A–E). In treatments with erastin or FAC, we observed increased cell death among De‐iPSC‐RPE cells and iPSC‐RPE cells, but few dead cells among iRPE cells (Figure 1F,G). We further induced ferroptosis in the three types of cells using the GPX4 inhibitor RSL3 and observed consistent results (Figure S1A, Supporting Information). To ascertain whether erastin, FAC, and RSL3 trigger cell death via ferroptosis, we treated the cells with deferoxamine (DFO), a ferroptosis inhibitor. The results showed that DFO was capable of rescuing the cells from death, providing conclusive evidence that ferroptosis was induced in cells by these compounds (Figure S1A–E, Supporting Information). These findings substantiate the superior ferroptosis resistance of iRPE cells versus De‐iPSC‐RPE cells and iPSC‐RPE cells.

Previous studies confirmed EMT‐related factor Zinc Finger E‐box‐Binding Homeobox 1 (ZEB1) promotes ferroptosis, and E‐Cadherin (CDH1) inhibits ferroptosis.^[^ ^19^ ^]^ Compared with De‐iPSC‐RPE cells, iRPE cells had lower ZEB1 expression and higher CDH1 expression (Figure S2A,B, Supporting Information). However, iRPE cells demonstrated higher ZEB1 expression and lower CDH1 expression than iPSC‐RPE cells (Figure S2A,B, Supporting Information). Both iRPE and iPSC‐RPE cells were in the epithelial state, yet only iRPE cells displayed stronger ferroptosis resistance than De‐iPSC‐RPE cells (Figure 1A–G). These results suggest that the enhanced ferroptosis resistance in iRPE is not attributable to maintaining epithelial state.

### 2.2 iRPE Cells Displayed Lower Levels of PE Molecular Species Compared with De‐iPSC‐RPE Cells and iPSC‐RPE Cells

**Figure 2:**
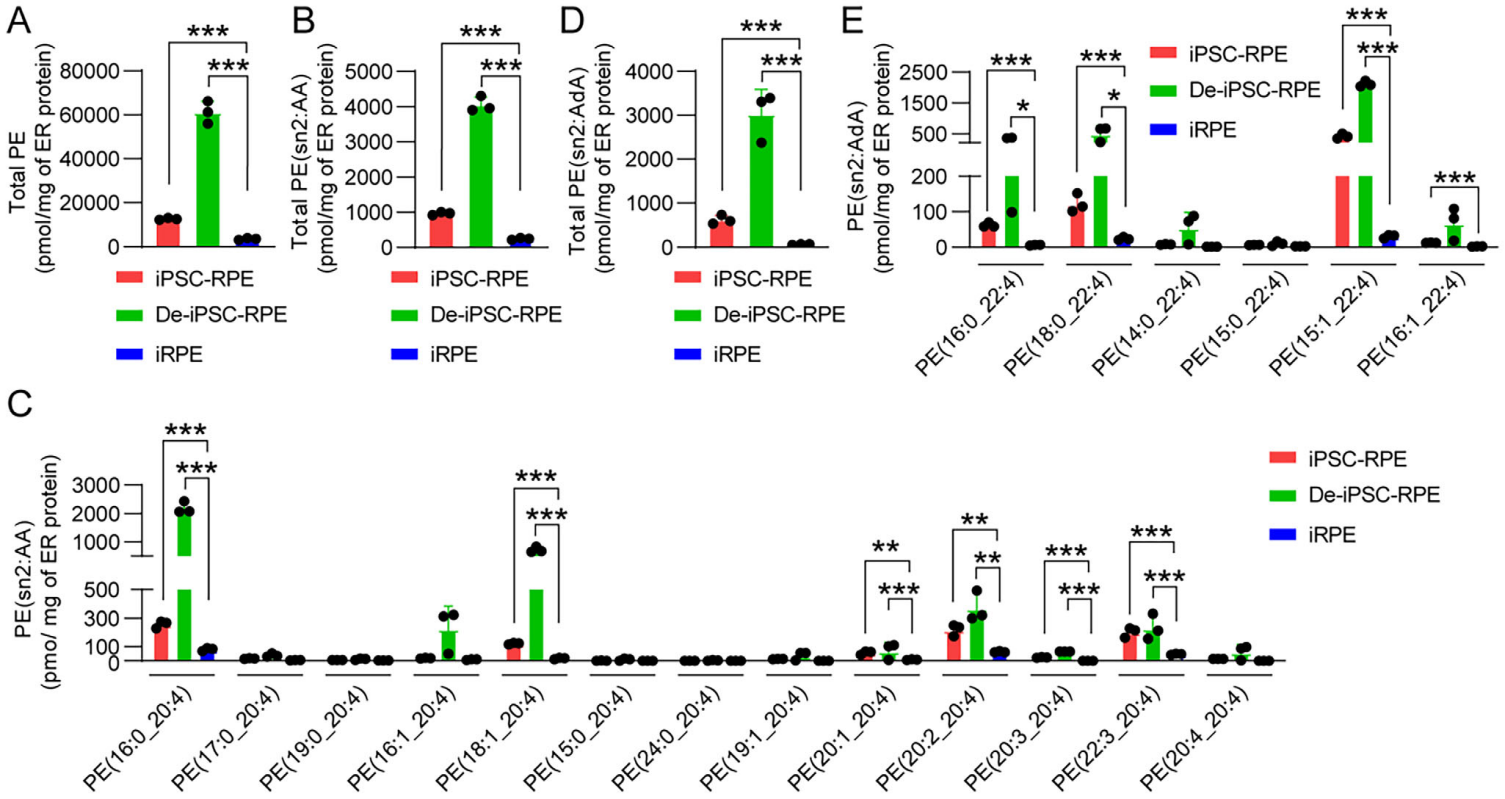
Lipidomics analysis of PE molecular species in iPSC‐RPE, De‐iPSC‐RPE, and iRPE cells. Lipids were extracted from the ER of iPSC‐RPE, De‐iPSC‐RPE, and iRPE cells and subjected to lipidomics analysis. The levels of PE molecular species were determined as pmol/mg of ER protein (*n* = 3). A) Total PE molecular species. B) Total PE molecular species with sn2:AA. C) Each PE molecular species with sn2:AA. D) Total PE molecular species with sn2:AdA. E) Each PE molecular species with sn2:AdA. Data are mean ± SD, *P < 0.05, **P < 0.01, ***P < 0.001 using one‐way ANOVA and post hoc Bonferroni's test. ER: endoplasmic reticulum, PE: Phosphatidylethanolamine, AA: arachidonic acid, AdA: adrenic acid.

Given that PE molecules are crucial to producing lipid peroxides that induce ferroptosis,^[^ ^13^ ^]^ we sought to determine whether the increased resistance of iRPE cells to ferroptosis is due to reduced PE levels. To this end, we isolated the ER, an essential organelle in the production of lipid peroxides in cells,^[^ ^13^, ^14^, ^21^ ^]^ and analyzed the levels and species of PE molecules. The results showed a significant reduction in the total PE levels in iRPE cells compared with De‐iPSC‐RPE cells and iPSC‐RPE cells (**Figure** **2**A). In addition, we examined PE molecules with sn2 side chains of AA or AdA (PE‐AA or PE‐AdA), the main sources of the lipid peroxides, and found significantly lower levels of the two types of PE molecules in iRPE cells (Figure 2B–E). Our findings suggest that the increased resistance of iRPE cells to ferroptosis is most likely due to the lower levels of PE molecular species.

To further confirm that lowering PE levels inhibits ferroptosis, we used meclizine to inhibit PCYT2, the rate‐limiting enzyme for PE synthesis.^[^ ^22^, ^23^ ^]^ A prior study reported that high concentrations (50 µM) of meclizine almost completely block PE synthesis.^[^ ^23^ ^]^ We found that 30 µm meclizine led to cell death, while 20 µM meclizine did not markedly affect the viability of De‐iPSC‐RPE cells (Figure S4A, Supporting Information). Therefore, we treated cells with 20 µM meclizine and found that it significantly lowered PE levels in the ER of De‐iPSC‐RPE cells (Figure S3A–E, Supporting Information) and enhanced ferroptosis resistance (Figure S4B–F, Supporting Information). This supports the notion that lowering PE levels can improve ferroptosis resistance. However, a critical level of PE is necessary for maintaining normal cellular functions, higher concentrations of meclizine likely exert more potent inhibition of PE synthesis, leading to cell death (Figure S4A, Supporting Information). Next, we treated iRPE cells with ethanolamine to elevate PE levels in the ER. At 2 mM, ethanolamine raised PE levels in the ER without affecting cell viability (Figures S3F–H and S4G, Supporting Information). Treating iRPE cells with 2 mM ethanolamine weakened their ferroptosis resistance (Figure S4H–L, Supporting Information). These findings confirm the relationship between PE levels in the ER and ferroptosis. Meclizine, originally developed as an antihistamine, inhibits PCYT2 only indirectly and exhibits dose‐dependent cytotoxicity at higher concentrations.^[^ ^23^ ^]^ Ethanolamine, on the other hand, requires multiple metabolic steps to elevate PE levels, resulting in low regulatory efficiency and poor specificity.^[^ ^24^ ^]^ Therefore, it is necessary to identify more specific and effective regulators of PE metabolism to further elucidate its role in ferroptosis.

### 2.3 PHOSPHO1 is a Suppressor of Ferroptosis in RPE Cells

**Figure 3:**
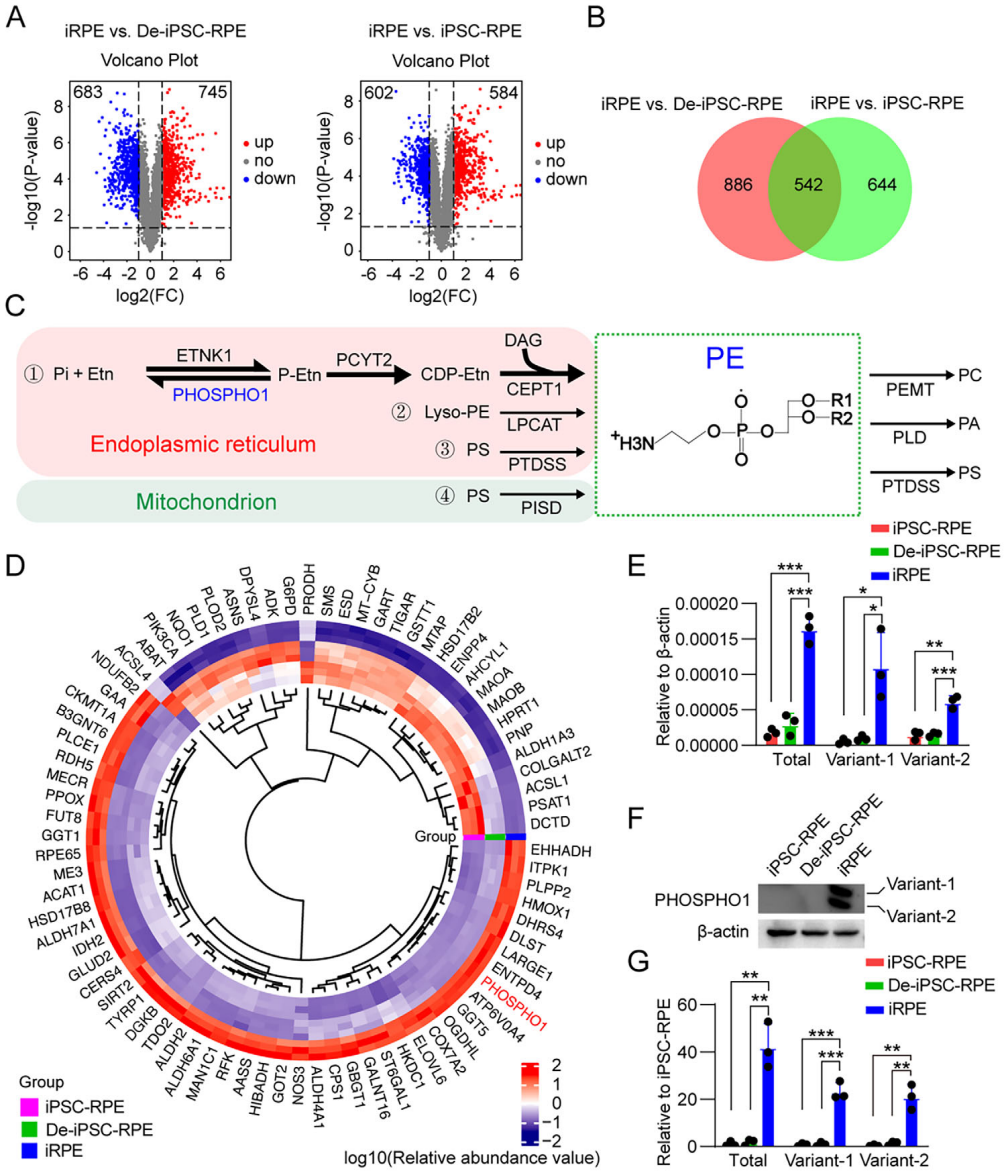
The expression level of PHOSPHO1 is higher in iRPE cells than in iPSC‐RPE cells and De‐iPSC‐RPE cells. A) Mass spectrometry was used to quantify the differentially expressed proteins (DEPs) in iRPE cells compared with De‐iPSC‐RPE cells and iPSC‐RPE cells. Volcano plot of 683 downregulated (blue) and 745 upregulated (red) DEPs (iRPE cells vs De‐iPSC‐RPE cells), and 602 downregulated (blue) and 584 upregulated (red) DEPs (iRPE cells vs iPSC‐RPE cells). B) The venn map demonstrated that 542 DEPs were upregulated in iRPE compared with De‐iPSC‐RPE cells and iPSC‐RPE cells. C) The diagram of the PE metabolic pathway. D) KEGG pathway analysis of 542 DEPs revealed that 80 DEPs were enriched in the metabolic pathway, and PE metabolism‐related enzyme PHOSPHO1 was highly expressed in iRPE cells. E) The expression levels of *PHOSPHO1* (two variants) in iPSC‐RPE cells, De‐iPSC‐RPE cells, and iRPE cells were determined by qRT‐PCR (*n* = 3). F,G) The expression levels of the two variants of PHOSPHO1 were determined and quantified by Western blotting (*n* = 3). Data are mean ± SD, *P < 0.05, **P < 0.01, ***P < 0.001 using one‐way ANOVA and post hoc Bonferroni's test.

To understand the causes of the decreased PE levels in iRPE cells, we used mass spectrometry (MS) analysis to quantify the levels of the key enzymes regulating PE metabolism in iRPE cells, De‐iPSC‐RPE cells, and iPSC‐RPE cells. We found 542 differentially expressed proteins (DEPs) in iRPE cells compared with De‐iPSC‐RPE cells and iPSC‐RPE cells (**Figure** **3**A,B). PE is primarily produced in the ER, and its synthesis is mostly mediated by the CDP‐ethanolamine pathway^[^ ^25^ ^]^ (Figure 3C). The 542 DEPs were further subjected to GO enrichment analysis, and 80 metabolism‐related DEPs were enriched (Figure 3D). Among the PE‐metabolizing related enzymes, PHOSPHO1 expression showed the most significant variation among the three types of cells (Figure 3D). Notably, iPRE cells were found to express two variants of *PHOSPHO1*, as validated by real‐time quantitative PCR (qRT‐PCR) and Western blotting (Figure 3E–G), and an analysis of previously published gene microarray data^[^ ^26^ ^]^ indicated that compared with the positive control *BEST1* gene, the expression level of *PHOSPHO1* is extremely low, almost non‐existent, and there is no difference in the RPE/choroid of normal individuals, dry AMD, and wet AMD patients (Figure S5, Supporting Information). Indeed, PHOSHPO1 was initially discovered to promote bone maturation.^[^ ^27^, ^28^ ^]^ We differentiated human umbilical cord mesenchymal stem cells (hUCMSCs) into osteocytes in vitro (Figure S6A, Supporting Information), and observed an upregulated expression of PHOSPHO1 in osteocytes compared with hUCMSCs (Figure S6B,C, Supporting Information). Furthermore, osteocytes demonstrated enhanced resistance to ferroptosis than hUCMSCs (Figure S6D,E, Supporting Information), indicating that PHOSPHO1 may have an inhibitory effect on ferroptosis.

**Figure 4:**
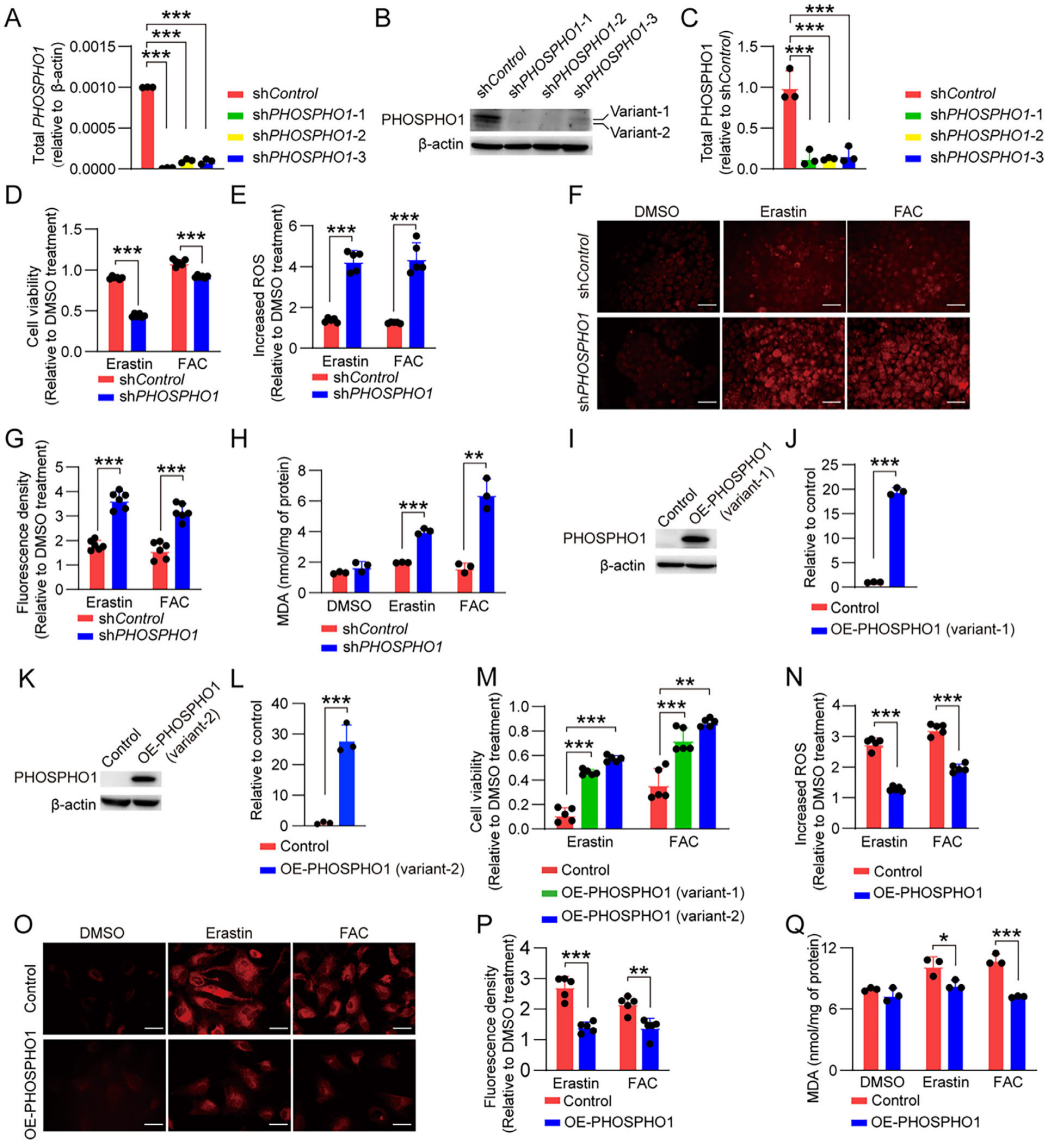
PHOSPHO1 inhibits ferroptosis. A) *PHOSPHO1* was knocked down in iRPE cells. The knockdown efficiency of *PHOSPHO1* was determined by qRT‐PCR (*n* = 3). B,C) The expression levels of the two variants of PHOSPHO1 after knockdown in iRPE cells were verified by Western blotting and quantitative analysis (*n* = 3). D–H) Ferroptosis was induced sh*Control*‐iRPE and sh*PHOSPHO1*‐iRPE cells by 30 µm erastin or 500 µM FAC, and DMSO treatment was used as a control, D) cell viability was analyzed by the CCK‐8 after being treated with erastin or FAC for 2 days (*n* = 6), E) intracellular ROS was assessed using the DCFH‐DA probe after being treated with erastin or FAC for 4 h (*n* = 5), F) intracellular Fe^2+^ levels under erastin or FAC treatment for 4 h were measured with the RhoNox‐1 fluorescent probe and G) quantified as fluorescence density relative to the DMSO treatment group (*n* = 6). H) The MDA was measured by MDA detection kit after being treated with erastin or FAC for 4 h and determined as nmol/mg of protein (*n* = 3). I–L) The variants 1 and 2 of PHOSPHO1 were overexpressed in De‐iPSC‐RPE cells, respectively, as verified by Western blotting and quantitative analysis, respectively (*n* = 3). M) Ferroptosis was induced in Control‐De‐iPSC‐RPE and OE‐PHOSPHO1‐De‐iPSC‐RPE cells by 30 µM Erastin or 500 µM FAC, (M) cell viability was analyzed by the CCK‐8 (*n* = 5), N) intracellular ROS was assessed using the DCFH‐DA probe after 4 h of 30 µM erastin or 500 µM FAC treatment (*n* = 5), O) intracellular Fe^2+^ levels were measured with the RhoNox‐1 fluorescent probe and P) quantified as fluorescence density relative to the DMSO treatment group (*n* = 5), Q) the MDA was measured by the MDA detection kit and determined as nmol/mg of protein (*n* = 3). Scale bar = 50 µm. Data are mean ± SD, *P < 0.05, **P < 0.01, ***P < 0.001 using unpaired two‐sided t‐tests in Figures. (j, l,) and one‐way ANOVA and post hoc Bonferroni's test for others.

To determine whether PHOSPHO1 could suppress ferroptosis, we knocked down *PHOSPHO1* in iRPE cells. Among the three shRNAs used, sh*PHOSPHO1*‐1 was more efficient at reducing the mRNA level of *PHOSPHO1* than the other two (**Figure** **4**A–C); hence, we selected sh*PHOSPHO1*‐1‐transfected iRPE (sh*PHOSPHO1*‐iRPE) cells for subsequent experiments. Compared with sh*Control*‐iRPE cells, sh*PHOSPHO1*‐iRPE cells were much less resistant to ferroptosis, as indicated by reduced viability and increased ROS, ferrous ions, and MDA under erastin or FAC stimulation (Figure 4D–H). In addition, after treatment with the ferroptosis inhibitor DFO, the resistance of sh*PHOSPHO1*‐iRPE cells to ferroptosis was partially restored compared to the untreated sh*Control*‐iRPE cells (Figure S1F–I, Supporting Information). Conversely, when the two variants of PHOSPHO1 were overexpressed in De‐iPSC‐RPE cells (Figure 4I–L), their viability after ferroptosis induction was higher than that of control cells transfected with an empty vector (Figure 4M). The De‐iPSC‐RPE cells with overexpressed PHOSPHO1‐variant‐2 were more resistant to ferroptosis than the cells with overexpressed PHOSPHO1‐variant‐1 (Figure 4M); therefore, De‐iPSC‐RPE cells with overexpressed PHOSPHO1‐variant‐2 (OE‐PHOSPHO1‐De‐iPSC‐RPE) were selected for subsequent experiments. We found that ferroptosis‐associated molecules ROS, ferrous ions, and MDA were significantly reduced in OE‐PHOSPHO1‐De‐iPSC‐RPE compared with control cells (Figure 4N–Q). These results confirm that PHOSPHO1 can inhibit ferroptosis. Further, by overexpressing PHOSPHO1 in iPSC‐RPE cells, we found reduced PE levels accompanied by increased resistance to ferroptosis (Figure S7A–G, Supporting Information), and overexpression of PHOSPHO1 did not alter epithelial structure and functional gene expression in iPSC‐RPE cells (Figure S7H–J, Supporting Information). To further illustrate that iRPE cells primarily inhibit ferroptosis through PHOSPHO1, we analyzed the expression of anti‐ferroptosis‐related proteins based on the data of MS protein profiling, and discovered that GPX4 and FSP1 were most abundant in iPSC‐RPE cells, the expression level of SLC7A11 in iRPE and De‐iPSC‐RPE cells was higher than that in iPSC‐RPE cells, and the expression level of FTH1 was lower in iRPE than that in De‐iPSC‐RPE cells and iPSC‐RPE cells (Figure S8, Supporting Information). However, pro‐ferroptotic protein Acyl‐CoA Synthetase Long‐Chain Family Member 4 (ACSL4),^[^ ^29^ ^]^ Cytochrome P450 Oxidoreductase (POR),^[^ ^30^ ^]^ and Lysophosphatidylcholine Acyltransferase 3 (LPCAT3)^[^ ^31^ ^]^ showed higher expression in De‐iPSC‐RPE cells, iPSC‐RPE cells, and iRPE cells, respectively (Figure S8, Supporting Information). These findings suggest that iRPE cells' enhanced ferroptosis resistance cannot be primarily attributed to differential expression of these proteins. Notably, the data of mass spectrometry demonstrated significantly higher PHOSPHO1 expression in iRPE cells compared to the De‐iPSC‐RPE cells and iPSC‐RPE cells. These results confirm that PHOSPHO1 inhibits ferroptosis in RPE cells, which is accompanied by a reduction in PE molecular species.

### 2.4 PHOSPHO1 Inhibits PE Synthesis to Suppress Ferroptosis

**Figure 5:**
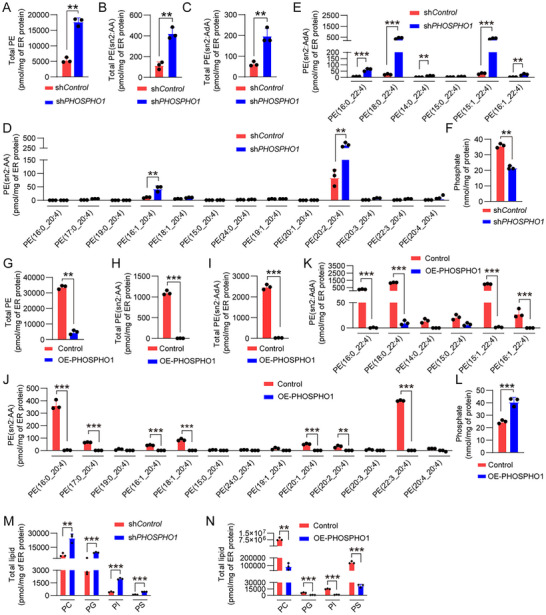
PHOSPHO1 reduces the levels of PE molecular species. A–E) PHOSPHO1 was knocked down in iRPE cells, and PE molecular species were detected and determined as pmol mg^−1^ of ER protein (*n* = 3);(A) Total PE molecular species in each group of cells were analyzed and determined as pmol/mg of ER protein (*n* = 3), (B) total PE molecular species with sn2:AA, (C) total PE molecular species with sn2:AdA, (D) the levels of PE molecular species with sn2:AA, (E) the levels of PE molecular species with sn2:AdA. F) Inorganic phosphate was detected by malachite green reagent in sh*Control*‐iRPE cells and sh*PHOSPHO1*‐iRPE cells. G–K) PHOSPHO1 was overexpressed in De‐iPSC‐RPE cells, and PE molecular species were detected and determined as pmol mg^−1^ of ER protein (*n* = 3); G) Total PE molecular species in each group of cells were analyzed and determined as pmol mg^−1^ of ER protein (*n* = 3), H) total PE molecular species with sn2:AA, I) total PE molecular species with sn2:AdA, J) the levels of PE molecular species with sn2:AA, K) the levels of PE molecular species with sn2:AdA. L) Inorganic phosphate was detected by malachite green reagent in Control‐De‐iPSC‐RPE cells and OE‐PHOSPHO1‐De‐iPSC‐RPE cells (*n* = 3). M) Total glycerophospholipid PC, PG, PI, and PS in sh*PHOSPHO1*‐iRPE cells (*n* = 3). N) Total PC, PG, PI, and PS in OE‐PHOSPHO1‐De‐iPSC‐RPE cells (*n* = 3). Data are mean ± SD, **P < 0.01, ***P < 0.001 using unpaired two‐sided t‐tests.

PHOSPHO1 is able to hydrolyze ethanolamine phosphate, the substrate for PE synthesis.^[^ ^32^ ^]^ To determine whether PHOSPHO1‐mediated ferroptosis resistance was achieved by altering PE metabolism, we analyzed the PE levels and demonstrated that the knockdown of PHOSPHO1 significantly increased total PE levels in the ER of iRPE cells (**Figure** **5**A). Specifically, the levels of PE species containing arachidonic acid (PE‐AA) and adrenic acid (PE‐AdA) were markedly elevated in PHOSPHO1‐knockdown iRPE cells (Figure 5B,C). A detailed analysis of individual PE‐AA species showed a significant increase in various molecular species in sh*PHOSPHO1*‐iRPE cells (Figure 5D), while the levels of PE‐AdA species were also significantly elevated (Figure 5E). Consistent with its enzymatic activity, PHOSPHO1 knockdown led to a significant reduction in cellular inorganic phosphate (Pi) levels (Figure 5F). Conversely, PHOSPHO1 overexpression in De‐iPSC‐RPE cells resulted in a significant reduction in total PE levels (Figure 5G), including both PE‐AA (Figure 5H) and PE‐AdA (Figure 5I). A detailed breakdown of PE‐AA species revealed a significant decrease in multiple molecular species in OE‐PHOSPHO1‐De‐iPSC‐RPE cells (Figure 5J), while the levels of PE‐AdA species were also reduced (Figure 5K). Additionally, the overexpression of PHOSPHO1 led to a notable increase in Pi levels, further confirming its enzymatic function (Figure 5L). We further examined the effects of PHOSPHO1 knockdown and overexpression on other major glycerophospholipids. The levels of phosphatidylcholine (PC), phosphatidylglycerol (PG), phosphatidylinositol (PI), and phosphatidylserine (PS) were significantly increased in sh*PHOSPHO1*‐iRPE cells (Figure 5M) and conversely reduced in OE‐PHOSPHO1‐De‐iPSC‐RPE cells (Figure 5N). These findings demonstrate that PHOSPHO1 inhibits ferroptosis in RPE cells by lowering PE molecular species and regulating other phospholipid components.

### 2.5 PHOSPHO1 Inhibits Ferroptosis by Weakening Autophagy

It has been shown that autophagy can promote ferroptosis, and its inhibition can prevent erastin‐induced ferroptosis in cells.^[^ ^15^, ^16^, ^33^ ^]^ To confirm the influence of autophagy on ferroptosis in RPE cells, we used serum starvation to promote autophagy and chloroquine to inhibit autophagy, then induced ferroptosis with either erastin or FAC. We observed that starvation augmented erastin‐ or FAC‐induced ferroptosis, while chloroquine inhibited ferroptosis (Figure S9, Supporting Information). These data confirm that autophagy promotes ferroptosis.

**Figure 6:**
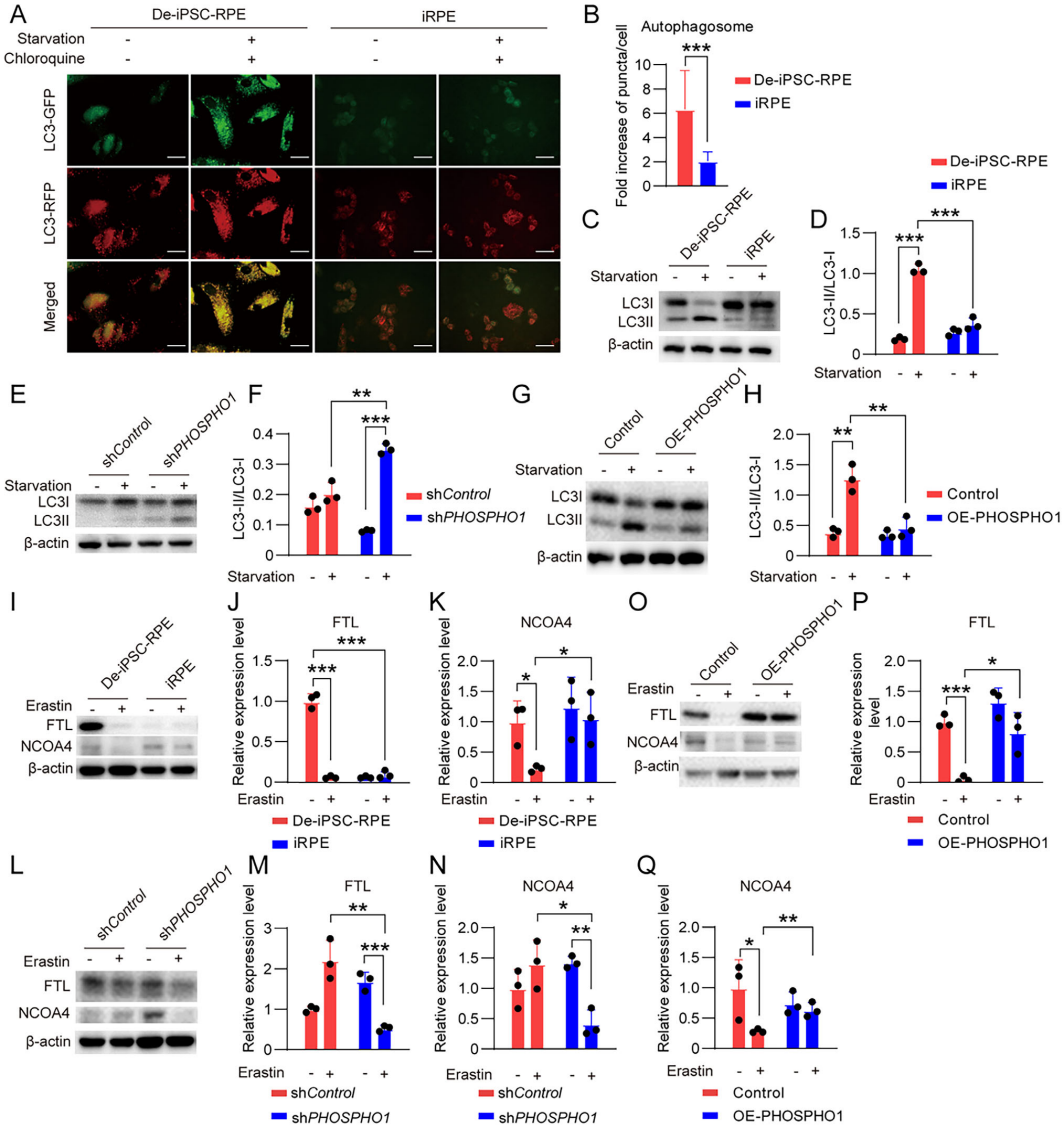
PHOSPHO1 weakens autophagy and ferritinophagy. A) The mRFP‐GFP‐LC3 plasmid was transfected into De‐iPSC‐RPE cells and iRPE cells that were cultured under starvation conditions to promote autophagy. Chloroquine was added into culture medium to inhibit lysosome activity and halt the quenching of GFP fluorescence, which made it easier to observe autophagosomes. The representative images demonstrated the formed autophagosome (yellow). B) The quantitative analysis of the fold increase in the number of autophagosomes in De‐iPSC‐RPE cells and iRPE cells (*n* = 50). C,D) The ratio of LC3II to LC3I in De‐iPSC‐RPE cells and iRPE cells under starvation conditions to induce autophagy was determined and quantified by Western blotting (*n* = 3). E,F) The ratio of LC3II to LC3I in sh*Control*‐iRPE cells and sh*PHOSPHO1*‐iRPE cells under starvation conditions was determined and quantified by Western blotting (*n* = 3). G,H) The ratio of LC3II to LC3I in Control‐De‐iPSC‐RPE cells and OE‐PHOSHPO1‐De‐iPSC‐RPE cells under starvation conditions was determined and quantified by Western blotting (*n* = 3). I–K) The levels of FTL and NCOA4 in De‐iPSC‐RPE cells and iRPE cells under erastin treatment were determined and quantified by Western blotting (*n* = 3). L–N) The levels of FTL and NCOA4 in sh*Control*‐iRPE cells and sh*PHOSPHO1*‐iRPE cells under erastin treatment were determined and quantified by Western blotting (*n* = 3). O–Q) The levels of FTL and NCOA4 in Control‐De‐iPSC‐RPE cells and OE‐PHOSHPO1‐De‐iPSC‐RPE cells under erastin treatment were determined and quantified by Western blotting (*n* = 3). Scale bar = 50 µm. Data are mean ± SD, *P < 0.05, **P < 0.01, ***P < 0.001 using unpaired two‐sided t‐tests for quantification of autophagosome and one‐way ANOVA and post hoc Bonferroni's test for the others.

The coupling of LC3‐I and PE to form the lipid‐bound LC3‐II and its localization to the phagophore is a key step in autophagosome formation,^[^ ^17^ ^]^ Once generated, LC3‐II is recruited to the membranes of nascent autophagosomes, facilitating their expansion and closure.^[^ ^34^ ^]^ Thus, reduced PE by PHOSPHO1 may prevent ferroptosis not only by directly reducing lipid peroxides but also by lowering autophagosome formation to weaken autophagy. To test this hypothesis, De‐iPSC‐RPE cells and iRPE cells were transfected with mRFP‐GFP‐LC3 plasmids, which encode a tandem fluorescent construct consisting of monomeric red fluorescent protein (mRFP), green fluorescent protein (GFP), and microtubule‐associated protein 1A/1B‐light chain 3 (LC3), to indicate autophagic flux. Cells were cultured under starvation conditions to promote autophagy. Chloroquine was added into culture medium to inhibit lysosome activity and halt the quenching of GFP fluorescence, which made it easier to observe autophagosomes. The results revealed that the fold change in the number of formed autophagosomes (yellow) in iRPE cells was less that in De‐iPSC‐RPE cells (Figure **6**A,B), confirming that the autophagic capacity of iRPE cells was weaker than that of De‐iPSC‐RPE cells. Additionally, the level of LC3‐II relative to LC3‐I is another accurate measurement of autophagic flux.^[^ ^29^ ^]^ The LC3II/LC3I ratio was significantly lower in iRPE than in De‐iPSC‐RPE cells under starvation conditions (Figure 6C,D), further indicating the weaker autophagic capacity of iRPE cells compared with De‐iPSC‐RPE cells. We then demonstrated that *PHOSPHO1* knockdown enhanced autophagic capacity in iRPE cells (Figure 6E,F), while PHOSPHO1 overexpression reduced autophagic capacity in De‐iPSC‐RPE cells (Figure 6G,H). These results indicate that PHOSPHO1 lowers PE levels to inhibit autophagy.

The mechanism by which autophagy promotes ferroptosis is proposed to involve ferritin degradation and increasing the concentration of free iron ions. The process is referred to as ferritinophagy and is mediated by nuclear receptor activator 4 (NCOA4).^[^ ^35^, ^36^ ^]^ We investigated the expression of ferritin light chain (FTL) and NCOA4 and found that their expression was reduced in De‐iPSC‐RPE cells under erastin stimulation (Figure 6I–K), indicative of ferritinophagy. However, in iRPE cells, the expression of FTL and NCOA4 did not change significantly (Figure 6I–K), substantiating a lower ferritinophagy capacity in iRPE cells than in De‐iPSC‐RPE cells. When *PHOSPHO1* was knocked down in iRPE cells, ferritinophagy was promoted (Figure 6L–N). Conversely, PHOSPHO1 overexpression inhibited ferritinophagy in De‐iPSC‐RPE cells (Figure 6O–Q). Together, these findings show that PHOSPHO1 weakens autophagy and ferritinophagy and enhances the ferroptosis resistance of iRPE cells.

### 2.6 PHOSPHO1 Inhibits Ferroptosis in RPE Cells in a Rat AMD Model

**Figure 7:**
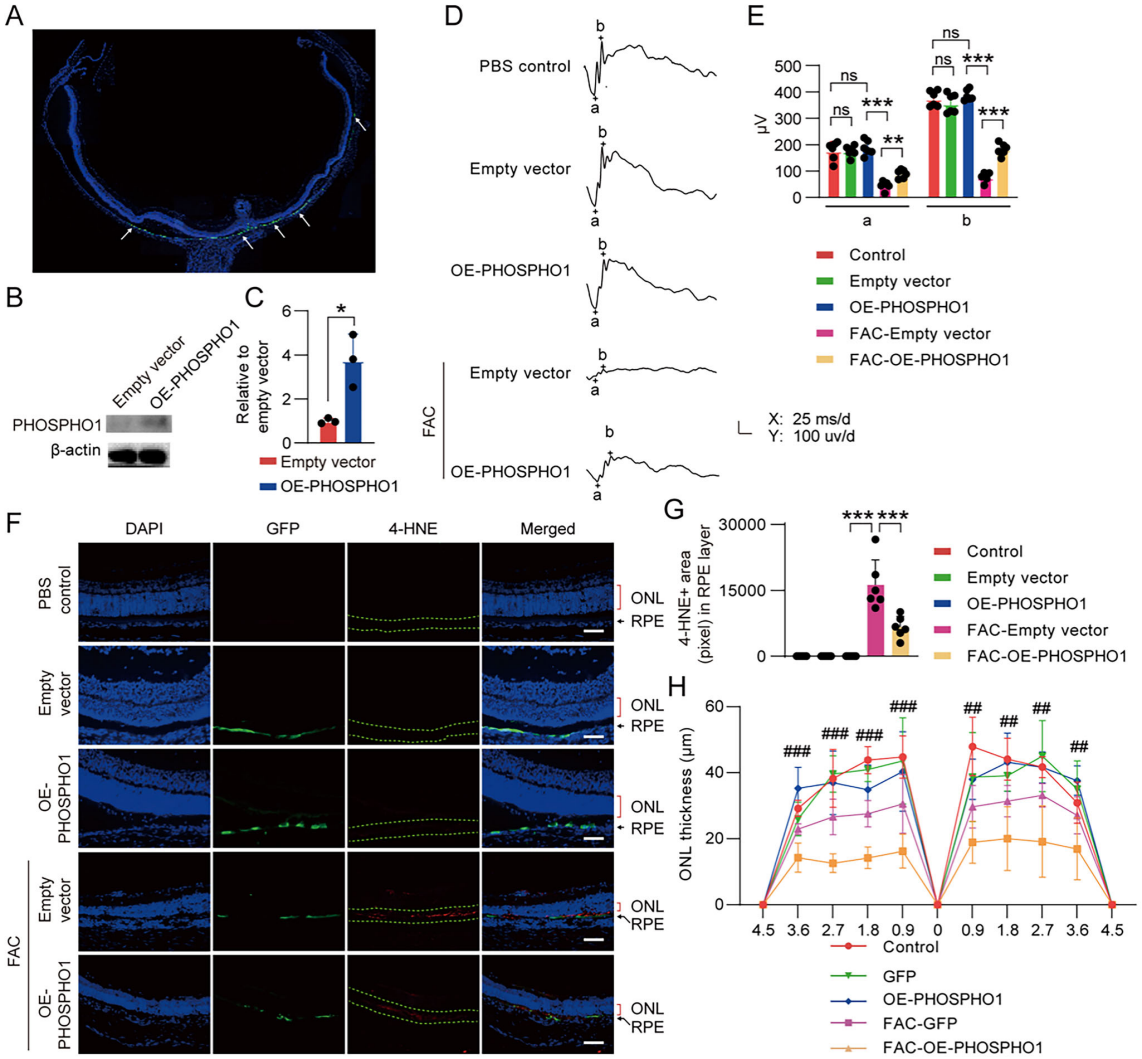
PHOSPHO1 inhibits ferroptosis in RPE cells in vivo. *PHOSPHO1* ‐expressing lentivirus was injected into the subretinal space of rats and then triggered RPE ferroptosis by intravitreous injection of FAC. Empty vector‐transfected lentivirus were used as control. A) The positively transfected lentivirus in RPE cells was indicated by the GFP labeling (arrow pointed). B,C) The RBCC was collected, and the overexpression of PHOSPHO1 in RPE cells was determined and quantified by Western blotting (*n* = 3). D) ERG waveforms were recorded on day 7 after FAC treatment (the calibration indicates 100 µV vertically and 25 ms horizontally). E) Quantitative analysis of ERG a‐ and b‐wave amplitudes (*n* = 6). F,G) 4‐HNE immunostaining in representative micrographs of retinal samples and the expression level of 4‐HNE was determined as 4‐HNE+ area (pixel) in RPE layer (RPE layer was between green dashed lines) (*n* = 6). H) Quantitative analysis of ONL thickness (*n* = 8). Scale bar = 50 µm. Data are mean ± SD, **P < 0.05, ***P < 0.001; ##*P* < 0.01, ###*P* < 0.001 OE‐PHOSPHO1 group compared with Empty vector group using unpaired two‐sided t‐tests for determination of PHOSPHO1 overexpression and one‐way ANOVA and post hoc Bonferroni's test for the others. ns: no significance.

To validate the ability of PHOSPHO1 to inhibit ferroptosis in RPE cells in vivo, we injected PHOSPHO1‐expressing lentivirus into the subretinal space of rats. RPE ferroptosis was then triggered by intravitreal injection of FAC.^[^ ^37^ ^]^ Retinal function was assessed one week later using electroretinograms (ERG). The lentivirus mainly infected RPE cells (**Figure** **7**A), To confirm that the PHOSPHO1 was overexpressed in RPE cells, the RPE‐Bruch's membrane‐choriocapillaris complex (RBCC) was collected, the overexpressed PHOSPHO1 was determined and quantified by Western blotting (Figure 7B,C). In addition, overexpression of PHOSPHO1 did not change the functions of RPE cells and neural retina, there was no difference in a‐, b‐wave amplitudes, and the outer nuclear layer (ONL) thickness between OE‐PHOSPHO1, Empty vector, and PBS control groups (Figure 7D–H). However, When treated with FAC to induce ferroptosis, we observed that FAC significantly diminished a‐ and b‐wave amplitudes (Figure 7D,E). However, in the PHOSPHO1‐overexpressed group, this reduction was less pronounced (Figure 7D,E). Histological staining showed a significant increase in the expression of 4‐Hydroxynonenal (4‐HNE) in the RPE layers after FAC injection indicating RPE cells undergoing ferroptosis (Figure 7F,G). However, overexpression of PHOSPHO1 reduced the level of 4‐HNE in RPE cells (Figure 7F,G). Subsequent analysis of ONL revealed iron overload‐induced ferroptosis in photoreceptor cells (Figure 7F,H), which resulted in a significant reduction in ONL thickness. In contrast, the ONL in the PHOSPHO1‐overexpressed group was significantly thicker (Figure 7F,H). Immunostaining further confirmed that overexpression of PHOSPHO1 in RPE cells not only inhibited ferroptosis in RPE cells, but also indirectly reduced ferroptotic photoreceptor cells (Figure S10, Supporting Information). Immunostaining of NCOA4 revealed that treatment with FAC enhanced NCOA4 staining intensity in RPE cells. Conversely, overexpression of PHOSPHO1 in RPE cells led to a decrease in NCOA4 expression level within these cells (Figure S11, Supporting Information).

To further verify the action of RPE cells on neural retinal cells, we collected iPSC‐RPE conditioned medium (iPSC‐RPE‐CM) and OE‐PHOSPHO1‐iPSC‐RPE‐CM to culture 661 W cells, a photoreceptor cell line. It was noted that the death of 661 W caused by erastin could be counteracted by both iPSC‐RPE‐CM and OE‐PHOSPHO1‐iPSC‐RPE‐CM (Figure S12A,B, Supporting Information). However, ferroptosis was directly induced in 661 W cells by both the conditioned media from erastin‐treated iPSC‐RPE and erastin‐treated OE‐PHOSPHO1‐iPSC‐RPE cells. Additionally, fewer ferroptotic 661 W cells were observed in the erastin‐treated OE‐PHOSPHO1‐iPSC‐RPE‐CM group (Figure S12C,D, Supporting Information), suggesting that ferroptotic RPE cells may produce and transfer lipid peroxide to photoreceptors, aggravating photoreceptor death, and PHOSPHO1 lowers PE levels to reduce the production of peroxide lipids, mitigating the damage of lipid peroxide to photoreceptors. These results collectively demonstrate that PHOSPHO1 can inhibit ferroptosis in RPE cells in vivo and indirectly mitigate the death of photoreceptors.

## 3 Discussion

This study demonstrates that high expression of PHOSPHO1 in iRPE cells lowered PE levels, reducing the generation of lipid peroxides. This reduction, coupled with simultaneously weakened autophagy, enhanced the resistance of iRPE cells to ferroptosis. PHOSPHO1 preventing ferroptosis was further validated in a rat model with FAC‐induced RPE cell ferroptosis.

PE molecular species are considered to be the key molecules in generating lipid peroxides that induce ferroptosis.^[^ ^13^, ^14^ ^]^ When oxidized, PE molecules directly disrupt the structure of the cell membrane, leading to rupture and necrosis.^[^ ^38^, ^39^ ^]^ The oxidized PE molecules can be further metabolized to MDA and 4‐HNE, both of which can alter the structure and function of proteins, and induce DNA damage, further promoting ferroptosis.^[^ ^38^, ^40^ ^]^ Previous studies have shown that PE molecules with AA or AdA at the sn‐2 position are substrates of lipid peroxide production.^[^ ^13^, ^14^ ^]^ However, in retinal cells where docosahexaenoic acid (DHA) is abundant and critical for photoreceptor and RPE cell homeostasis,^[^ ^41^, ^42^ ^]^ DHA‐derived peroxides can also cause the onset of cell lesions in the RPE cells.^[^ ^43^, ^44^ ^]^ Thus, unlike cells of other organs, PE‐DHA in RPE cells may also be a significant substrate for the generation of lipid peroxides. Furthermore, the binding of PE to LC3I to form LC3II is involved in generating autophagosomes.^[^ ^17^ ^]^ We confirmed that reducing PE can attenuate autophagic capacity. However, the specific type of PE molecules involved in autophagosome formation is still unclear.

There are four PE synthesis pathways in eukaryotic cells: i) the CDP‐ethanolamine pathway; ii) acylation of lysophosphatidylethanolamine to PE by lysophosphatidylethanolamine acyltransferase; iii) head group exchange between phosphatidylserine and ethanolamine; and iv) decarboxylation of phosphatidylserine in mitochondria, catalyzed by phosphatidylserine decarboxylase (PSD).^[^ ^25^ ^]^ The first three occur in the ER, and the fourth in the mitochondria. The CDP‐ethanolamine and PSD pathways are the two primary pathways for PE biosynthesis. The other two pathways, lyso‐PE acylation and head group exchange, minimally contribute to PE synthesis.^[^ ^25^ ^]^ The CDP‐ethanolamine pathway is more efficient than the PSD pathway. Hence, most PE molecules in cells are synthesized by the CDP‐ethanolamine pathway in the ER.^[^ ^25^ ^]^ Previous studies showed that lipid peroxides mainly originate from the ER,^[^ ^13^, ^14^, ^21^ ^]^ and PE involved in LC3II synthesis also originate from the ER.^[^ ^45^, ^46^ ^]^ Thus, detecting PE levels in the ER will more directly elucidate the connection between PE and ferroptosis.

Previous studies have demonstrated that iron ion level is elevated in AMD patients^[^ ^4^, ^47^ ^]^ and that long‐term iron treatment for anemia is associated with AMD features.^[^ ^1^ ^]^ Intravitreal injection of FAC, as revealed by HE staining, caused significant RPE damage and thinning of the outer nuclear layer.^[^ ^48^ ^]^ These results are similar to those observed in our previously used sodium iodate model^[^ ^18^ ^]^ and RCS rat model,^[^ ^49^ ^]^ indicating that intravitreal injection of FAC can induce AMD‐like symptoms. These findings imply that iron ion‐induced ferroptosis is one of the causes of RPE degeneration in the pathogenesis of AMD. Age‐related decline in the ability of RPE cells to counteract oxidative stress leads to increased ROS and organelle damage. Autophagy is then activated to remove damaged molecules and diseased organelles, like mitochondria, to maintain the homeostasis of RPE cells.^[^ ^50^, ^51^ ^]^ However, enhanced autophagy degrades ferritin via the ferritinophagy pathway, increasing intracellular iron ion concentrations and activating the ferroptosis signaling pathway.^[^ ^1^ ^]^ Our findings reveal that iRPE cells have lower autophagic activity and higher ferroptosis resistance than De‐iPSC‐RPE cells. Increased autophagy promotes ferroptosis, aligning with observations that PHOSPHO1's suppression of autophagy contributes to the inhibition of ferroptosis. We further found that intravitreal injection of FAC in vivo increased the expression of NCOA4, whereas overexpression of PHOSPHO1 decreased NCOA4 expression. These results were inconsistent with our in vitro findings. A possible explanation for this discrepancy is that in vitro experiments utilized erastin to stimulate cells, which promotes ferritinophagy and increases intracellular iron ion concentration. In contrast, in vivo experiments involved intravitreal injection of FAC, directly elevating iron ion levels. Consequently, the increase in NCOA4 expression might represent a passive response to the elevated iron levels. Autophagy acts as a double‐edged sword under oxidative stress. Thus, autophagy‐activating ferroptosis could be a critical mechanism of RPE cell death in AMD. After transplanting into the subretinal space of the rat AMD model, iRPE cells demonstrated stronger therapeutic capacity than iPSC‐RPE cells, possibly due to their enhanced resistance to ferroptosis.^[^ ^18^ ^]^ Despite reduced autophagic activity, iRPE cells maintain a requisite level of autophagy for normal functioning.

PHOSPHO1 can hydrolyze ethanolamine phosphate and choline phosphate into Pi, ethanolamine, and choline.^[^ ^32^ ^]^ Thus, it was initially discovered to be crucial for bone formation by elevating cellular Pi.^[^ ^52^ ^]^ In addition, the enzyme is essential for terminal erythropoiesis.^[^ ^53^ ^]^ PHOSPHO1 lowers PE and PC levels by reducing the substrates (ethanolamine phosphate and choline phosphate) for their synthesis.^[^ ^53^ ^]^ We also confirmed that PHOSPHO1 was able to reduce the levels of PE and PC. However, we found that PHOSPHO1 could reduce the levels of PG, PI, and PS, it might be achieved through indirect regulation. Moreover, PHOSPHO1 has been recognized as a key metabolic biomarker in the tumorigenesis of diffuse large B‐cell lymphoma.^[^ ^54^ ^]^ We also discovered that PHOSPHO1 is highly expressed in recurrent OS (Figure S2, Supporting Information), which indicates that PHOSPHO1 may inhibit the ferroptosis of tumor cells. Given PE's involvement in the synthesis of GPI‐anchored proteins,^[^ ^55^ ^]^ the increased expression of PHOSPHO1 in tumor cells may reduce GPI‐anchored proteins on the cell membrane, thereby limiting the presence of some tumor antigens on the cell membrane. The vital role of PE in cellular processes means that its complete inhibition leads to cell death. This was evident when high doses of meclizine, an inhibitor of a critical enzyme in PE synthesis, resulted in cell death. PHOSPHO1 does not inhibit PE synthesis entirely due to its indirect role in the PE metabolism pathway, allowing PE levels to remain within viable ranges. Therefore, iRPE cells with elevated PHOSPHO1 expression demonstrated enhanced resistance to ferroptosis while retaining typical RPE cell functioning.^[^ ^18^ ^]^ Besides reducing PE levels, PHOSPHO1 can also increase intracellular Pi concentration, which may prevent ferroptosis by inhibiting the PP2A/Src/ACSL4 signaling pathway.^[^ ^56^, ^57^ ^]^

It is believed that the pathophysiology of AMD results from the functional impairment of RPE cells, which in turn causes the death of photoreceptor cells.^[^ ^58^ ^]^ RPE cells provide as trophic support for photoreceptor cells. We found normally functioning RPE cells were able to prevent photoreceptor cell death through paracrine actions, whereas ferroptotic RPE cells produced compounds that promote 661 W cells death. These compounds are most likely lipid peroxides, which are transported to photoreceptor cells by extracellular vesicles, speeding up the death of photoreceptor cells. This implies that throughout the pathophysiology of AMD, the functionally impaired RPE cells not only show decreased trophic support of photoreceptor cells, but may also actively cause photoreceptor cell death via paracrine actions. We attempted to use optical coherence tomography to identify the structure of the retina, but we could not obtain a clear image, possibly because the large size of rat lens, when we did subretinal injection with a needle through vitreous chamber, the needle may slightly touch and damage the lens, weakening the transparency of the lens. To prevent damage to the lens, we will undertake subretinal injection through the scleral route in the future.

**Figure 8:**
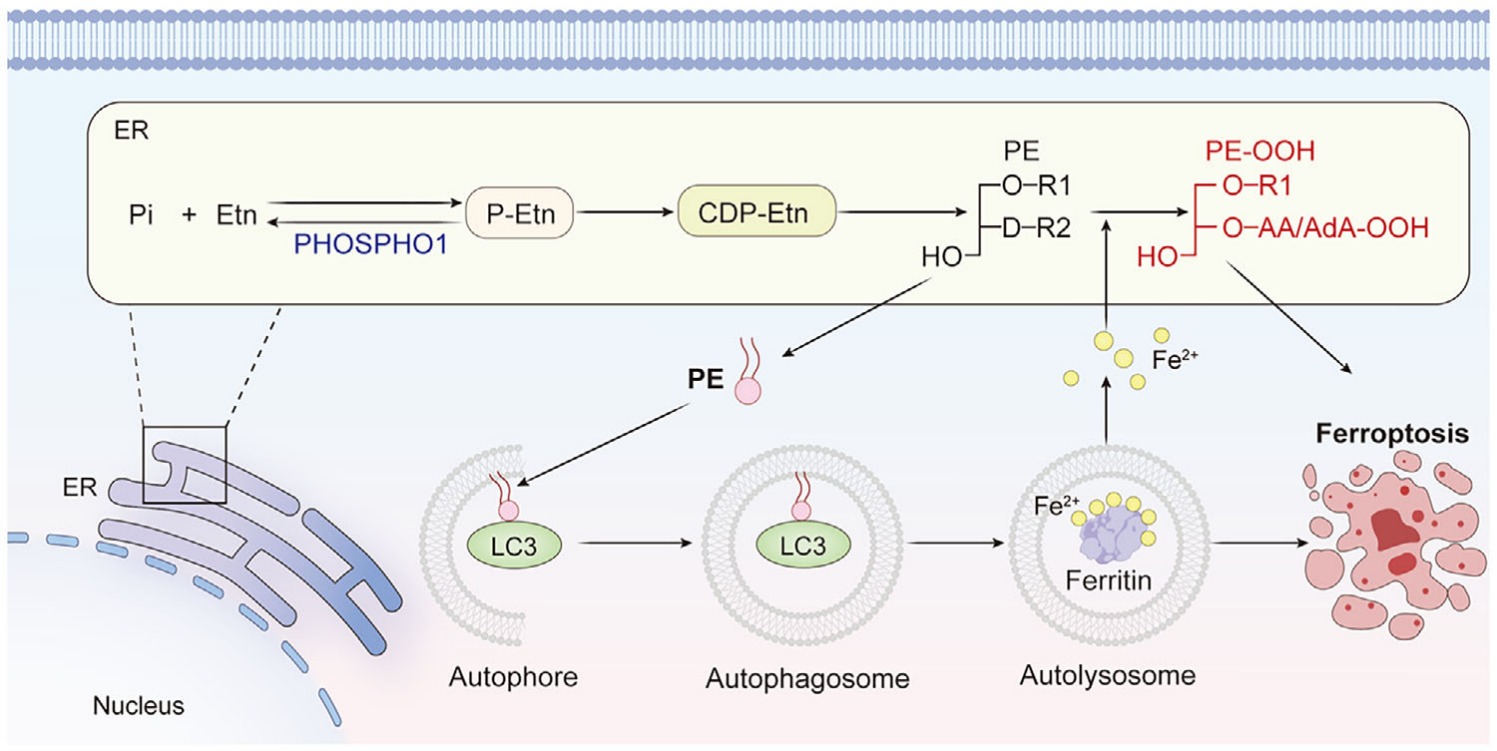
Diagram showing the processes by which PHOSPHO1 prevents ferroptosis. By decreasing the substrate P‐Etn for PE synthesis, PHOSPHO1 lowers the level of PE and thus directly reduces the formation of lipid peroxides. Lowering the level of PE can also prevent the creation of autolysosomes, reduce the production of free irons, and consequently decrease the amount of lipid peroxides produced indirectly. Etn: ethanolamine, P‐Etn: phosphoethanolamine, PE: phosphatidylethanolamine, PE‐OOH: phosphatidylethanolamine peroxide, ER: endoplasmic reticulum.

In summary, this study demonstrated that iRPE cells derived from De‐iPSC‐RPE cells were more resistant to ferroptosis. Our investigations reveal that iRPE cells expressed high levels of the PHOSPHO1 enzyme, which reduces PE levels in the ER. This reduction not only decreased the generation of lipid peroxides but also weakened autophagic ability to prevent ferroptosis in RPE cells (**Figure** **8**). The role of PHOSPHO1 in preventing ferroptosis was further validated in a rat model with FAC‐induced RPE cell ferroptosis. Our findings also confirmed that a moderate reduction in PE levels was sufficient to inhibit ferroptosis, whereas complete blockage of PE synthesis resulted in cell death. Therefore, PE can be used as a target to prevent ferroptosis, and a strategy to precisely modulate PE metabolism to prevent ferroptosis in RPE cells is promising for the treatment of AMD. In addition, PHOSPHO1 may also be used as a target for treatment of cancers.

## 4 Experimental Section

### Cell Cultures

According to previous reports,^[^ ^18^, ^59^ ^]^ iPSC‐RPE cells were cultured in DMEM/F12, 10% knockout serum replacement (KSR), 1% nonessential amino acids, 2 mm glutamine, 50 U ml^−1^ penicillin and 50 mg ml^−1^ streptomycin (all from Invitrogen, Carlsbad, CA), and 10 mM nicotinamide (Sigma, St. Louis, MO) in dishes precoated with Matrigel (Corning, Charlotte, NC, USA). De‐iPSC‐RPE cells and iRPE cells were cultured in DMEM/F12 supplemented with 10% FBS (Applied StemCell, California, USA) and 50 U ml^−1^ penicillin, 50 mg ml^−1^ streptomycin. Human umbilical cord mesenchymal stem cells (hUCMSCs) were obtained from the Eastern Union Stem Cell and Gene Engineering Co., Ltd. (China) and cultured in DMEM/F12 containing 10% FBS at 37 °C and 5% CO_2_. For osteogenesis, the hUCMSCs were maintained in osteogenic induction medium (Shanghai QiDa Biotechnology Co., Ltd, Shanghai, China) and cultured for two weeks. Osteocytes were examined for Alkaline Phosphatase (AKP) activity by the Alkaline Phophatase Staining Kit (Solarbio, Beijing, China). 661 W cells were purchased from SUNNCELL (Wuhan, China), cells were cultured in DMEM/F12 containing 10% FBS.

### Cell Viability

Cell viability assay was performed following the protocol of the CCK‐8 assay kit (Beyotime, Shanghai, China). In brief, cells were seeded in 96‐well culture plates at a density of 1500 cells per well in DMEM/F12 containing 10% KSR (for iPSC‐RPE cells) or 10% FBS (for De‐iPSC‐RPE cells and iRPE cells) and different dosages of erastin (0 to 100 µM, MedChemExpress, Shanghai, China) or ferric ammonium citrate (FAC, 0 to 1600 µm, MedChemExpress). Two days later, CCK‐8 reagent was added to each well and incubated for 2 h at 37 °C. Using a microplate reader, the optical absorbance was measured at 450 nm.

### Measurement of ROS Production

ROS was detected by the Reactive Oxygen Species Assay Kit (Beyotime) according to the manufacturer's instructions. Briefly, cells were cultured in a 96‐well culture plate and treated with 30 µM Erastin or 500 µM FAC for 4 h. Cell culture medium was removed, and 50 µL DMEM/F12 containing 10 µM DCFH‐DA was added into each well. Cells were cultured for 60 min at 37 °C, and the fluorescence intensity was measured by a multifunctional microplate reader (Synergy H1, BioTek, USA) with an excitation wavelength of 488 nm.

### MDA Measurement

Malondialdehyde (MDA) was detected by the MDA detection kit (Beyotime) according to the manufacturer's instructions. Briefly, cells were cultured in a 6‐well culture plate and treated with 30 µM Erastin or 500 µM FAC for 4 h. Cells were lysed with lysis buffer, and protein concentration was measured by the bicinchoninic acid assay (BCA) method. Hundred microliters lysed sample was mixed with 0.2 mL TBA working solution and incubated at 100 °C for 15 min. The absorbance value was measured by a multifunctional microplate reader (Synergy H1) with a wavelength of 532 nm. The MDA concentration was determined as nmol per mg of protein.

### Fe^2+^ Measurement

Cells were cultured in a 48‐well culture plate and treated with 30 µM erastin or 500 µM FAC for 4 h. Cell culture medium was removed, and DMEM/F12 containing 1 µM RhoNox‐1 (MedChemExpress) was added into each well. Cells were incubated at 37 °C and 5% CO_2_ for 1 h. The intracellular ferrous iron was stained with RhoNox‐1. The samples were then examined by fluorescence microscope (Olympus IX73, Tokyo, Japan). Ferrous iron concentration was determined by fluorescence density.

### Lipid Profiling Analysis

ER was extracted from 2 × 10^7^ cells of each sample with the Minute ER Enrichment Kit (Invent Biotechnologies, Beijing, China). One milliliter lipid extraction solution (including the internal standard mixture, MetWare Biotechnology Co., Ltd., Wuhan, China) was used to extract the lipid molecules from the ER by sonication. After centrifugation at 12 000 x g at 4 °C for 10 min, the 200 µL supernatant was used for mass spectrometry (MS) analysis. Samples (2 µL) were separated on a Thermo accucore C30 column (2.6 µm, 2.1 mm × 100 mm, Sunnyvale, CA, USA) with a flow rate of 0.35 mL min^−1^ and a column temperature of 45 °C. The mobile phases consisted of a mixture of acetonitrile/water (60:40, v/v) (A) and a mixture of acetonitrile/isopropanol (10:90, v/v) (B), both containing 0.1% formic acid and 10 mmol L^−1^ ammonium formate. The elution gradient was set stepwise as follows: 0 min, A/B (80:20, V/V); 2 min, A/B (70:30, V/V); 4 min, A/B (40:60, V/V); 9 min, A/B (15:85, V/V); 14 min, A/B (10:90, V/V); 15.5 min, A/B (5:95, V/V); 17.3 min, A/B (5:95, V/V); 17.5 min, A/B (80:20, V/V); 20 min, A/B (80:20, V/V). MS analysis was performed on a triple quadrupole‐linear ion trap mass spectrometer (QTRAP), API 6500+. The electrospray ionization source operation parameters were as follows: ion source, turbo spray; source temperature 500 °C; ion spray voltage 5500 V for positive ion mode and ‐4500 V for negative ion mode; ion source gas I, gas II, and curtain gas were set at 45, 55, and 35 psi, respectively. The quantitative analysis of lipid profiling was performed by utilizing multiple reaction monitoring analysis at MetWare Biotechnology Co., Ltd., Wuhan, China.

### Protein Profiling Analysis

Cells were harvested and lysed in lysis buffer followed by protein quantification with the BCA method. Fifty micrograms proteins from each sample were used for trypsin digestion and then for Tandem Mass Tag labeling. High‐performance liquid chromatography (HPLC) analysis was performed on an 1100 HPLC System (Agilent) using an Agilent Zorbax Extend‐C18 column (5 µm, 150 mm × 2.1 mm). Mobile phases A (2% acetonitrile in HPLC water) and B (90% acetonitrile in HPLC water) were used for the gradient. Tryptic peptides were separated at a fluent flow rate of 300 µL min^−1^ and monitored at 210 nm. The separated peptides were lyophilized for MS analysis.

MS analysis was performed by a Q‐Exactive HF mass spectrometer (Thermo, USA) equipped with a Nanospray Flex source (Thermo, USA). Samples were loaded and separated by a C18 column (50 cm × 75 µm) on an EASY‐nLCTM 1200 system (Thermo, USA). Full MS scans were acquired in the mass range of 350–1500 m/z with a mass resolution of 60 000, and the AGC target value was set at 3e6. The 20 most intense peaks in MS were fragmented with higher‐energy collisional dissociation, with a collision energy of 32. MS/MS spectra were obtained with a resolution of 45 000, with an AGC target of 2e5, and a maximum injection time of 80 ms. The Q‐Exactive HF dynamic exclusion was set for 30.0 s. The quantitative analysis of protein profiling was performed by Oebiotech, Shanghai, China.

The protein expression levels were calculated by the relative abundance value. Log2 fold change (FC) of relative abundance value (iRPE cells vs De‐iPSC‐RPE cells), (iRPE cells vs iPSC‐RPE cells), and (iPSC‐RPE vs De‐iPSC‐RPE) was used to identify differentially expressed proteins (DEPs). Only those proteins indicating |log2FC| > 1 and adjusted p < 0.05 were regarded as DEPs.

### qRT‐PCR

Total RNA was extracted and reverse transcription was performed using Primescript RT Master Mix kit (Takara, Shiga, Japan). Real‐time quantitative PCR (qRT‐PCR) was performed in a Chromo4 instrument cycler (Bio‐Rad, Hercules) using Superreal Premix plus kit (Tiangen Biotech, Beijing, China). PCR amplification was carried out with the following cycling parameters: denaturation at 95 °C for 5 min, followed by 40 cycles of 95 °C for 30 s, 60 °C for 30 s. Primer sequences (Synthesized by Sangon Biotech Co., Ltd., Shanghai, China) were listed in Table S1 (Supporting Information).

### Western Blotting

The cells were lysed by RIPA buffer containing protease and phosphatase inhibitor (Sigma). The protein extracts (20 µg per sample) were separated by 10% SDS‐PAGE gels, and transferred onto polyvinylidene difluoride membranes (Millipore, Bedford, MA). After being blocked with 5% BSA in TBST for 1 h, membranes were incubated with primary antibodies against PHOSPHO1 (Santa Cruz Biotechnology), LC3, E‐cadherin (Cell signaling technology), NCOA4 (Absin), FTL, ZEB1, CRALBP, and β‐ACTIN (Proteintech), TYRP‐1(Abcam) for 12 h at 4 °C, followed by incubation with corresponding secondary antibodies for 1 h at room temperature. The blots were visualized with a chemiluminescence imaging system (Tanon 5200, Shanghai, China) and quantified with Image J software (Version 1.48v). Antibodies are listed in Table S2 (Supporting Information).

### Analysis of Microarray Data

The microarray data of human RPE/choroid, which includes 7 normal samples, 7 dry AMD samples, and 7 wet AMD samples (with the age of all the individuals being over 75) were obtained from the GEO (https://www.ncbi.nlm.nih.gov/geo/) database (GSE29801). The relative level of *PHOSPHO1* in each group was compared, *BEST1* was used as positive control.

### Generation of Lentiviruses to Knockdown *PHOSPHO1* in iRPE Cells

Lentiviral pLVX‐shRNA2‐ZsGreen1 (Takara) vector was used to prepare lentiviruses. The packaging plasmids were psPAX2 and pMD2.G. The targeting sequences of the three shRNA for *phospho1* were included in Table S3 (Supporting Information). HEK293FT cells (ATCC, Research Triangle Park, NC, USA) were transfected with vectors. Individual supernatants containing virus were harvested at 48 h and used to infect iRPE cells. The positively transfected cells were sorted by fluorescence‐activated cell sorting (FACS) based on Zoanthus sp. Green (ZsGreen) expression. The reduced expression of target genes at transcript level was determined by qRT‐PCR. The most efficient shRNA was selected from the three for subsequent experiments.

### PHOSPHO1 Overexpression

For generating lentivirus, human cDNAs of *phospho1‐variant1*, *phospho1‐variant2*, and rat cDNA of *phospho1* were obtained by PCR amplification from iRPE cells and rat testis and cloned into lentiviral pLVX‐mCMV‐ZsGreen1‐Puro vector (Takara). The packaging plasmids were psPAX2 and pMD2.G. HEK293FT cells were seeded at a density between 5.0–7.0 × 10^4^ cells cm^−2^ and transfected by Lipofectamine 2000 (Invitrogen) with each vector. Individual supernatants containing virus were harvested at 48 h post‐transfection and filtered with a 0.45 µm PVDF membrane (Millipore, Boston). iPSC‐RPE cells and De‐iPSC‐RPE cells were plated in 10‐cm culture dishes at 5.0 × 10^6^ or 5.0 × 10^5^ cells per dish, respectively. The next day, cells were infected with viruses. The positively transfected cells were sorted by FACS based on ZsGreen expression. The expression of PHOSPHO1 was determined by qRT‐PCR and Western blotting.

### Phosphate Assay

Phosphate in cells was quantified by the Malachite Green Phosphate Detection Kit (Beyotime) according to the manufacturer's instructions. Briefly, cells were collected with 0.25% trypsin/EDTA, and cell membranes were disrupted by ultrasound. After centrifuging at 10 000 x g for 20 min, the supernatant was collected, and the supernatant and phosphate standard were added into a 96‐well culture plate (200 µL per well). Seventy microliters Malachite green reagent working solution was added into each well and incubated at room temperature for 30 min. The absorbance value (O.D. value) at the 630 nm wavelength was measured.

### mRFP‐GFP‐LC3 Cell Model Construction

Autophagy indicator mRFP‐GFP‐LC3 (HB‐AP210‐0001) was purchased from HanBio (Shanghai, China). Adenoviral infection was performed according to the manufacturer's instructions. iRPE cells and De‐iPSC‐RPE cells, grown to 70% confluence, were incubated with the adenoviruses in medium for 6 h, and then in complete DMEM/F12 medium containing 4 µg mL^−1^ puromycin for 48 h. Based on different pH stability, the fluorescent signal of GFP could be quenched under the acidic condition inside the lysosome, while the mRFP fluorescent signal has no significant change. Autophagic flux can be observed or determined by evaluating the number of GFP and mRFP puncta per cell in merged images.

### Autophagic Flux and Ferritinophagy Determination

OE‐PHOSPHO1‐De‐iPSC‐RPE and sh*PHOSPHO1*‐iRPE cells were cultured under starvation conditions (DMEM/F12 + 1% FBS) to promote autophagy. Ferroptosis was induced by 30 µM erastin. The expression levels of LC3I, LC3II, FTL, and NCOA4 were analyzed by Western blotting. Autophagic flux was indicated by LC3II/LC3I and ferritinophagy was determined by the expression levels of FTL and NCOA4.

### Establishment of the In Vivo RPE Ferroptosis Model

Six‐week‐old SD female rats (Laboratory Animal Center of Tongji University) were used in this study. The experimental rats were randomly assigned into five groups: control, empty vector, OE‐PHOSPHO1, FAC‐Empty vector, and FAC‐OE‐PHOSPHO1 groups. In vivo RPE ferroptosis model was established according to the previous report.^[^ ^37^ ^]^ Briefly, the six‐week‐old SD rats were anesthetized with 2% sodium pentobarbital. A channel was created by inserting a 30‐gauge needle, behind the limbus, into the vitreous chamber. A 33‐gauge needle was inserted into the subretinal space of the central retina, and 3 µL lentivirus carrying the rat *phospho1* gene was injected. The control eyes received an injection of lentivirus carrying an empty vector. Three days later, a 33‐gauge needle was inserted into the vitreous chamber, and 2 µL FAC (1 mM) was injected into the vitreous chamber to induce ferroptosis. Those rats were sacrificed with an overdose of sodium pentobarbital one week later, eyes were collected and fixed for preparation of cryosections. Prior to the commencement of the study, rats appeared healthy and demonstrated normal activity levels. Rats showing signs of illness or abnormal behavior were initially excluded from the study. Animals were excluded from the analysis only if they encountered complications not related to the RPE ferroptosis model, such as infections or physical abnormalities not associated with the induced RPE ferroptosis. No blinding method was used for assessing outcome.

### ERG Examination

Following cell transplantation, ERG examination was performed one week later after FAC injection with AVES‐2000 electrophysiological apparatus (Kanghuaruiming S&T, Chongqing, China) as described previously.^[^ ^49^ ^]^ A stimulus intensity of 6.325 e‐2 cd•s/m^2 was used to elicit and record photoreceptor responses.

### Retina Structure Assessment

The SD rats were sacrificed with an overdose of sodium pentobarbital one week later after FAC injection. The eyeballs were removed immediately and fixed in 4% PFA. The embedded tissues were sectioned (10 µm thickness) along the vertical meridian of the eye. The samples were collected and used to assess retinal degeneration and RPE ferroptosis. Nuclei in the sectioned tissue were counterstained with 4,6‐diamidino‐2‐phenylindole dihydrochloride (DAPI, Sigma). The degree of retinal degeneration was assessed by the thicknesses (µm) of retinal outer nuclear layer (ONL), which were measured at 8 different points within both the nasal and temporal hemispheres.

### Immunostaining

For immunofluorescence analysis, cells or cryosections from eyes were permeabilized with 0.1% Triton X‐100 (Sigma) for 10 min, washed with PBS, and then blocked with 3% bovine serum albumin (BSA, Sigma) in PBS. The samples were incubated with the primary antibodies against CRALBP, TYRP1, Rhodopsin, ZO‐1, NCOA4, and 4‐HNE (ThermoFisher) overnight at 4 °C. They were then washed three times with PBS, followed by incubation with the fluorescent secondary antibodies (Invitrogen) overnight at 4 °C. DAPI was used to indicate the nucleus. The samples were then examined by a fluorescence microscope (Olympus IX73, Tokyo, Japan). Antibody is listed in Table S2 (Supporting Information).

### iPSC‐RPE Conditioned Medium

iPSC‐RPE cells were cultured in DMEM/F12 without or with 20 µM erastin. Two days later, the medium was removed and the cells were washed with DMEM/F12 to remove residual erastin. The cells were cultured in DMEM/F2 for further 2 days. The medium was collected and used for iPSC‐RPE‐CM.

### Calcein/PI Staining

Cells were cultured in 96‐well culture plate. For Calcein/PI staining, cells were cultured in 100 µL of calcein AM/PI working solution (Beyotime) at 37 °C for 1 h. The samples were then examined by a fluorescence microscope (Olympus IX73).

### Statistical Analysis

All values were expressed as the mean ± SD. Data were analyzed using GraphPad Prism 9 software (GraphPad Software, San Diego, CA, USA). All statistical analyses were performed using Student's t‐test, or one‐way ANOVA and post hoc Bonferroni's test. Statistical significance was set at *P* < 0.05.

### Ethics Approval

All the experimental rats had free access to water and standard rodent chow and were housed in a specific pathogen‐free environment with a temperature of 25 °C, a humidity of 40–70%, and a 12 h light‐dark cycle. All animal procedures were performed according to the institutional guidelines and the Guide for the Care and Use of Laboratory Animals issued by the NIH and the guidelines of the animal experimentation ethics committee of Tongji University (Approved NO. TJAA09623202), and in accordance with the Association for Research in Vision and Ophthalmology Statement for the use of Animals in Ophthalmic and Vision Research.

## Conflict of Interest

The authors declare no conflict of interest.

## Author Contributions

Y.B., L.L., G.‐T.X., H.T., Z.C., X.Z., M.M.L., and Q.O. contributed equally to this work and Z.C., X.Z., M.M.L., and Q.O. are the co‐first authors. Z.Y.C. performed conceptualization, investigation, methodology, validation, formal analysis, visualization, wrote the original draft. X.M.Z., M.M.L., and Q.J.O. performed investigation, methodology, validation, wrote the original draft. X.Y.W., Z.Z.Z., Q.S., and Q.W. performed investigation and methodology. Z.W., J.Y.X., C.X.J., F.R.G., J.W., and J.F.Z. performed investigation. J.P.Z. and X.L.J. performed investigation and reviewed and edited the draft. Y.L.B., G.T.X., and L.X.L. performed funding acquisition and reviewed and edited the draft. H.B.T. performed conceptualization, funding acquisition, supervision, project administration, validation, reviewed and edited the draft. All authors read and approved the final manuscript.

## Supporting information



Supporting Information

## Data Availability

The data that support the findings of this study are openly available in ProteomeXchange Consortium at http://proteomecentral.proteomexchange.org, reference number 47825.
