# Supplementary material for: PHOSPHO1 Suppresses Ferroptosis in Retinal Pigment Epithelial Cells by Reducing the Levels of Phosphatidylethanolamine Molecular Species
Source: Adv Sci (Weinh). 2025 May 21;12(28):2505359. doi: 10.1002/advs.202505359 (PMC12302541; doi:10.1002/advs.202505359)
Supplement: Supplementary file 1 — Supporting Information [file ADVS-12-2505359-s001.docx]

Supporting Information

**PHOSPHO1 suppresses ferroptosis in retinal pigment epithelial cells by reducing the levels of phosphatidylethanolamine molecular species**

*Zhiyang Chen^1, †^, Xiaoman Zhu^1, †^, Michael Mingze Lu^1, †^, Qingjian Ou^1, 2, †^, Xueying Wang^1^, Zhenzhen Zhao^1^, Qi Shen^1^, Qian Wang^1^, Zhe Wang^3^, Jing-Ying Xu^1^, Caixia Jin^1^, Furong Gao^1^, Juan Wang^1^, Jingfa Zhang^4^, Jieping Zhang^1, 2^, Xiaoliang Jin^5^, Yanlong Bi^1, *^, Lixia Lu^1, *^, Guo-Tong Xu^1, *^, Haibin Tian^1,2, *^*

1 Department of Ophthalmology of Tongji Hospital and Laboratory of Clinical and Visual Sciences of Tongji Eye Institute, School of Medicine, Tongji University, Shanghai 200065, China;

2 Department of Physiology and Pharmacology, School of Medicine, Tongji University, Shanghai 200092, China;

3 Department of Physiology, College of Basic Medical Sciences, Naval Medical University, Shanghai 200433, China;

4 The International Eye Research Institute of the Chinese University of Hong Kong (Shenzhen), Shenzhen 518000, China.

5 Department of Ophthalmology, Ninth People’s Hospital, Shanghai Jiao Tong University School of Medicine, Shanghai 200025, China

* Address correspondence to these authors at the Department of Ophthalmology of Tongji Hospital and Laboratory of Clinical and Visual Sciences of Tongji Eye Institute, School of Medicine, Tongji University, 389 Xincun Road, Shanghai 200065 China; E-mail: [biyanlong@tongji.edu.cn](mailto:biyanlong@tongji.edu.cn), lulixia@tongji.edu.cn, xuguotong@tongji.edu.cn, and tianhb@tongji.edu.cn. ^*^Yanlong Bi, Lixia Lu, Guo-Tong Xu, and Haibin Tian are co-corresponding authors, who contributed equally to this study.

† Zhiyang Chen, Xiaoman Zhu, Michael Mingze Lu, and Qingjian Ou are the co-first authors who contributed equally to this study.

### Authors and Affiliations

**Department of Ophthalmology of Tongji Hospital and Laboratory of Clinical and Visual Sciences of Tongji Eye Institute, School of Medicine, Tongji University, Shanghai 200065, China**

Z. Chen, X. Zhu, M. Lu, Qi. Ou, X. Wang, Z. Zhao, Q. Shen, Q. Wang, J. Xu, C. Jin, F. Gao, J. Wang, J. Zhang, Y. Bi, L. Lu, G. Xu, H. Tian

**Department of Physiology and Pharmacology, School of Medicine, Tongji University, Shanghai 200092, China**

J. Zhang, Q. Ou, H. Tian

**Department of Physiology, College of Basic Medical Sciences, Naval Medical University, Shanghai 200433, China**

Z. Wang

**The International Eye Research Institute of the Chinese University of Hong Kong (Shenzhen), Shenzhen 518000, China**

J. Zhang

**Department of Ophthalmology, Ninth People’s Hospital, Shanghai Jiao Tong University School of Medicine, Shanghai 200025, China**

X. Jin

# Supporting Figures

**Supplementary Fig. 1**


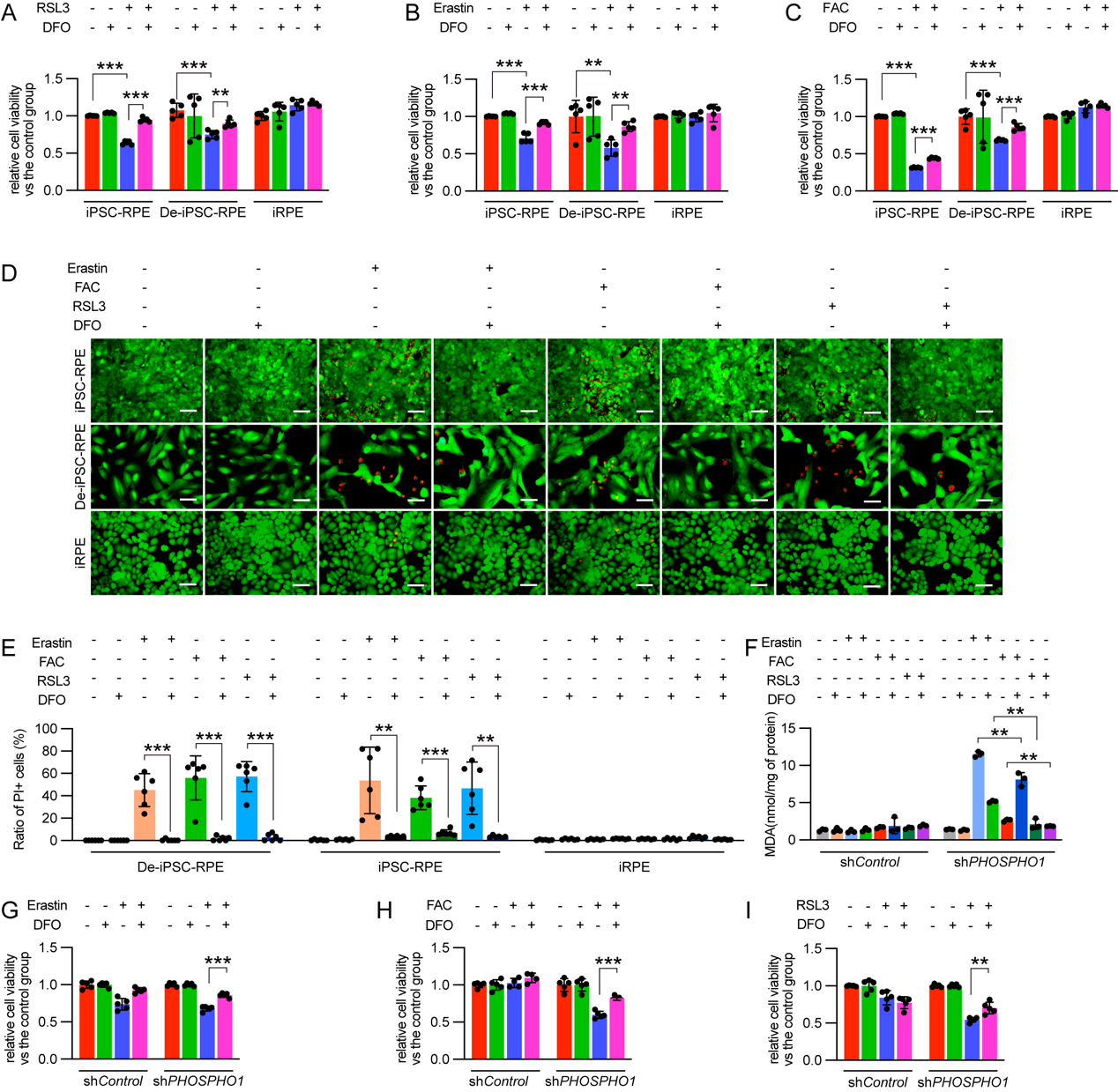
 **Supplementary Fig. 1.** **DFO Rescues Ferroptosis. (A-C)** DFO rescues ferroptosis in iPSC-RPE and De-iPSC-RPE cells, with cell viability analyzed using the CCK-8 assay, **(A)** Ferroptosis induced by 20 μM RSL3 was rescued by 100 μM DFO in iPSC-RPE and De-iPSC-RPE cells (n = 5). **(B)** Ferroptosis induced by 30 μM erastin was rescued by 100 μM DFO in iPSC-RPE and De-iPSC-RPE cells (n = 5), **(C)** Ferroptosis induced by 500 mM FAC was rescued by 100 μM DFO in iPSC-RPE and De-iPSC-RPE cells (n = 5), **(D)** Dead cells were detected using Calcein/PI staining, and **(E)** the proportion of PI+ cells was quantified (n = 6). Scale bar = 50 μm. **(F)** MDA levels were measured using a detection kit and expressed as nmol/mg protein. (n = 3). **(G-I)** DFO rescues ferroptosis in sh*PHOSPHO1*-iRPE cells, with cell viability analyzed using the CCK-8 assay. **(G)** Ferroptosis induced by 30 μM erastin was rescued by 100 μM DFO in sh*PHOSPHO1*-iRPE cells (n = 5). **(H)** Ferroptosis induced by 500 mM FAC was rescued by 100 μM DFO in sh*PHOSPHO1*-iRPE cells (n = 5). **(I)** Ferroptosis induced by 20 μM RSL3 was rescued by 100 μM DFO in sh*PHOSPHO1*-iRPE cells (n = 5). Data are mean ± SD, *P < 0.05, **P < 0.01 using one-way ANOVA and post hoc Bonferroni’s test or Student’s t-test.

**Supplementary Fig. 2**


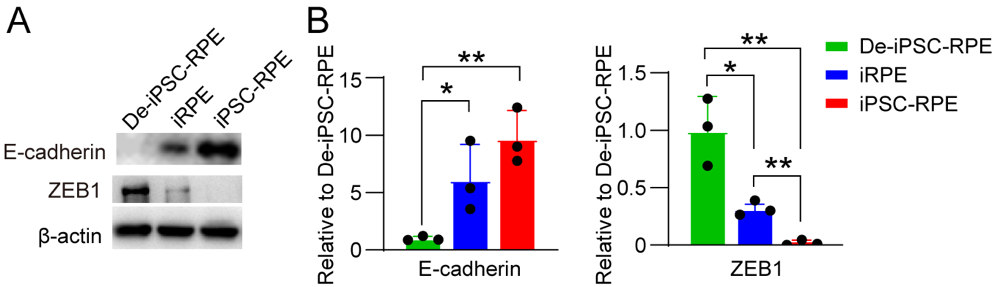


**Supplementary Fig. 2. The expression of E-cadherin and ZEB1 in De-iPSC-RPE cells, iRPE cells, and iPSC-RPE cells.** (**A** and **B**) The expression levels of E-cadherin and ZEB1 in De-iPSC-RPE cells, iRPE cells, and iPSC-RPE cells were determined by (**A**) Western blotting and (**B**) quantitative analysis (n = 3). Data are mean ± SD, *P < 0.05, **P < 0.01 using one-way ANOVA and post hoc Bonferroni’s test.

**Supplementary Fig. 3**


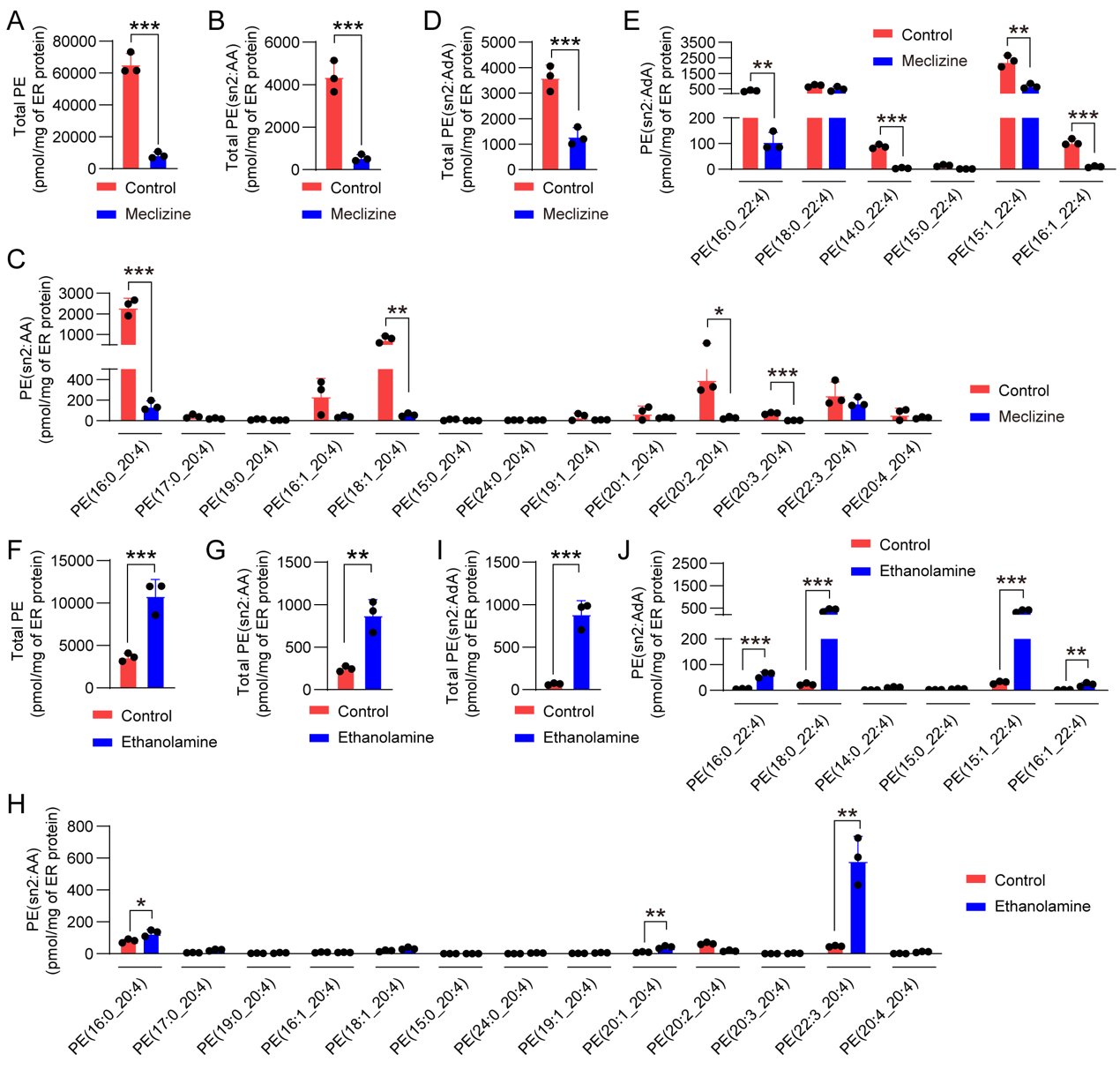


**Supplementary Fig. 3. Meclizine and ethanolamine change the levels of PE molecular species.** (**A-E**) De-iPSC-RPE cells were treated with 20 μM meclizine for 2 days, PE molecular species were detected and determined as pmol/mg of ER protein (n = 3); (**A**) total PE molecular species, (**B**) total PE molecular species with sn2:AA, (**C**) each PE molecular species with sn2:AA, (**D**) total PE molecular species with sn2:AdA, (**E**) each PE molecular species with sn2:AdA. (**F-J**) iRPE cells were treated with 2 mM ethanolamine for 2 days, PE molecular species were detected and determined as pmol/mg of ER protein (n = 3); (**F**) total PE molecular species, (**G**) total PE molecular species with sn2:AA, (**H**) each PE molecular species with sn2:AA, (**I**) total PE molecular species with sn2:AdA, (**J**) each PE molecular species with sn2:AdA. Data are mean ± SD, *P < 0.05, **P < 0.01, ***P < 0.001 using unpaired two-sided t-tests.

**Supplementary Fig. 4**


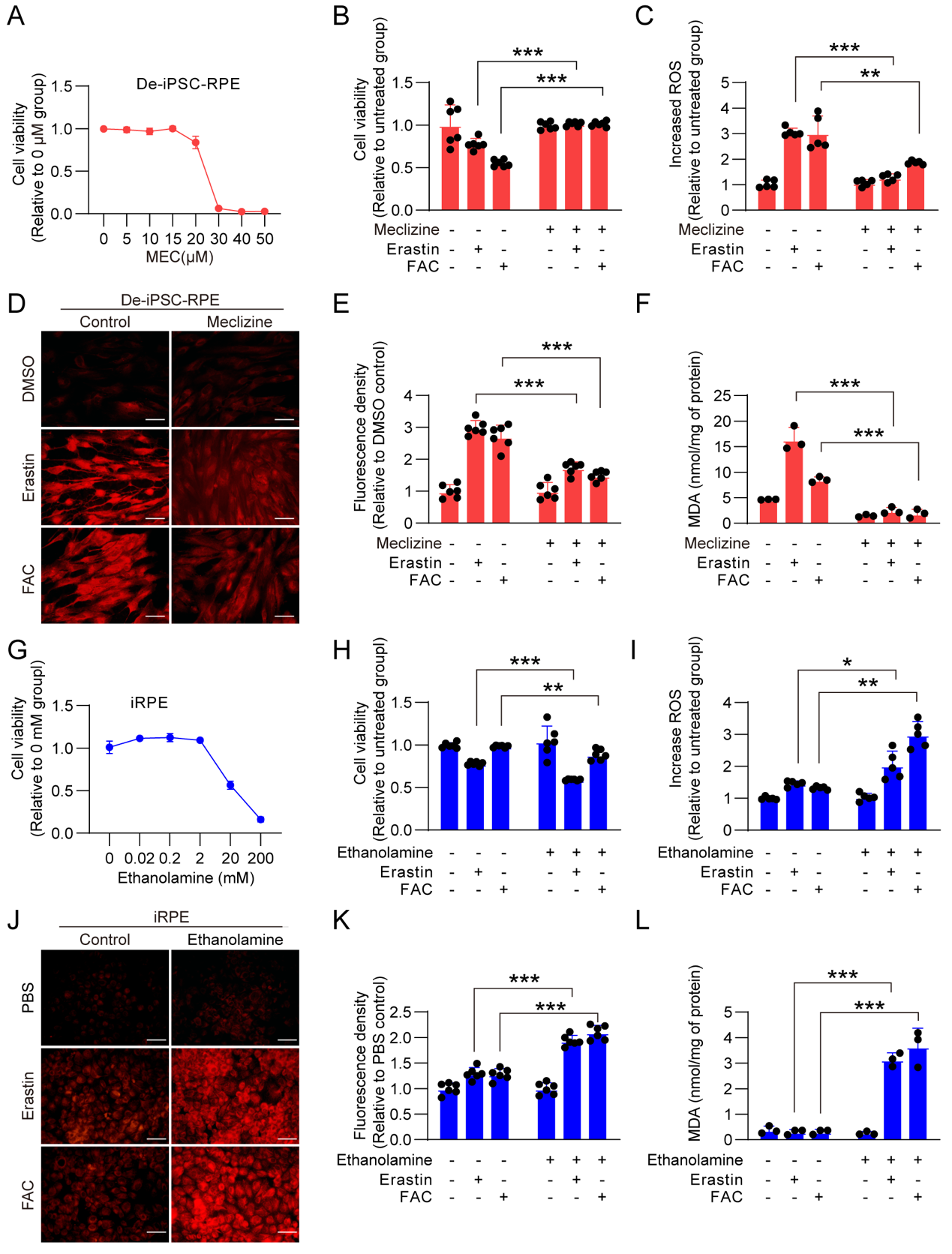


**Supplementary Fig. 4. Meclizine and ethanolamine regulate ferroptosis.** De-iPSC-RPE cells were treated with meclizine, and iRPE cells were treated with ethanolamine. (**A**) Cell viability of De-iPSC-RPE cells treated with different doses of meclizine was analyzed by the CCK-8 kit; 20 μM meclizine did not reduce cell viability significantly, thus this dose was used in subsequent experiments (n = 6). (**B-F**) De-iPSC-RPE cells were pretreated with 20 μM meclizine for 2 days and then treated with 30 μM erastin or 500 μM FAC, ferroptosis was detected; (**B**) cell viability was analyzed by the CCK-8 kit (n = 6), (**C**) intracellular ROS was measured with the DCFH-DA probe (n = 5), (**D**) intracellular Fe^2+^ level was measured with the RhoNox-1 fluorescent probe and (**E**) quantified as fluorescence density relative to the DMSO control (n = 6), (**F**) the MDA was measured by the MDA detection kit and determined as nmol/mg of protein (n = 3). (**G**) Cell viability of iRPE cells treated with different doses of ethanolamine was analyzed by CCK-8 kit, 2 mM ethanolamine did not reduce cell viability, thus this dose was used in subsequent experiments (n = 6). (**H-L**) De-iPSC-RPE cells were pretreated with 2 mM ethanolamine for 2 days and then treated with 30 μM erastin or 500 μM FAC, ferroptosis was detected; (**H**) cell viability was analyzed by CCK-8 kit (n = 6), (**I**) intracellular ROS was measured with the DCFH-DA probe (n = 5), (**J**) intracellular Fe^2+^ level was measured with the RhoNox-1 fluorescent probe and (**K**) quantified as fluorescence density relative to DMSO control (n = 6), (**L**) MDA was measured by the MDA detection kit and determined as nmol/mg of protein (n = 3). Scale bar = 50 μm. Data are mean ± SD, *P < 0.05, **P < 0.01, ***P < 0.001 using one-way ANOVA and post hoc Bonferroni’s test.

**Supplementary Fig. 5**

**
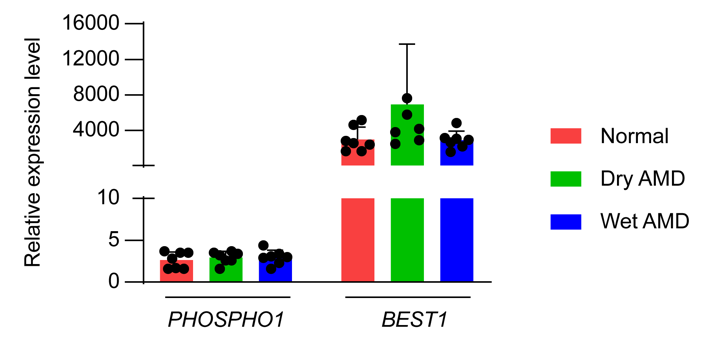
**

**Supplementary Fig. 5. The expression of *PHOSPHO1* in human RPE/choroid.** The expression level of *PHOSPHO1* was confirmed by the gene microarray data from healthy normal people, dry AMD, and wet AMD patients, *BEST1* was used as a reference marker to indicate relative expression levels*.* (n = 7).

**Supplementary Fig. 6**


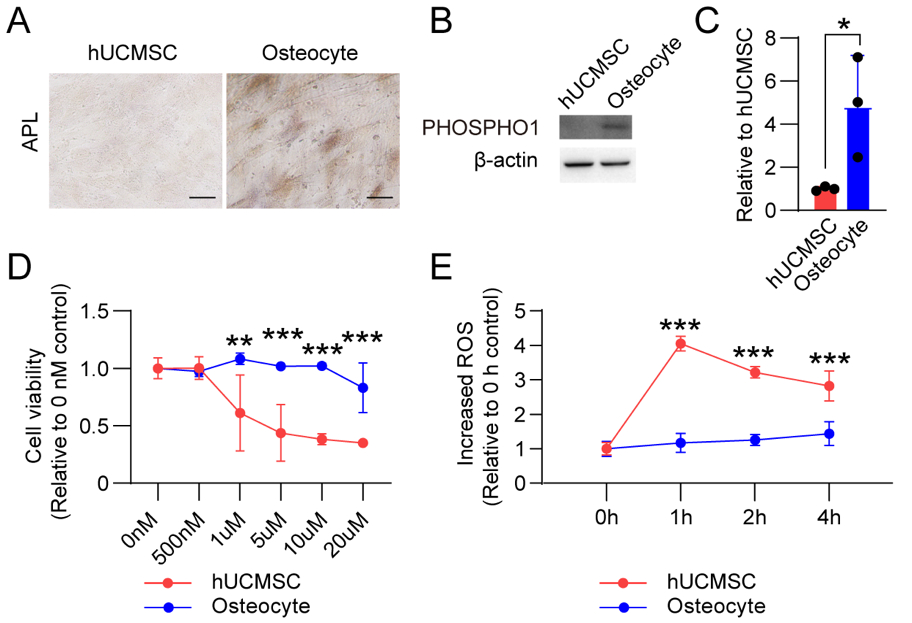


**Supplementary Fig. 6. Osteocytes derived from hUCMSCs demonstrate enhanced resistance to ferroptosis.** (**A**) hUCMSCs were differentiated into osteocytes, which showed increased alkaline phosphatase (APK) activity. (**B**) and (**C**) The expression level of PHOSPHO1 was determined by Western blotting and quantitative analysis (n = 3). (**D**) Cells were subjected to 30 μM erastin treatment, and cell viability was analyzed by CCK-8 kit (n = 6). (**E**) Intracellular ROS was assessed using the DCFH-DA probe (n = 6). Data are mean ± SD, *P < 0.05, **P < 0.01, ***P < 0.001 compared with hUCMSC using unpaired two-sided t-tests.

**Supplementary Fig. 7**

**
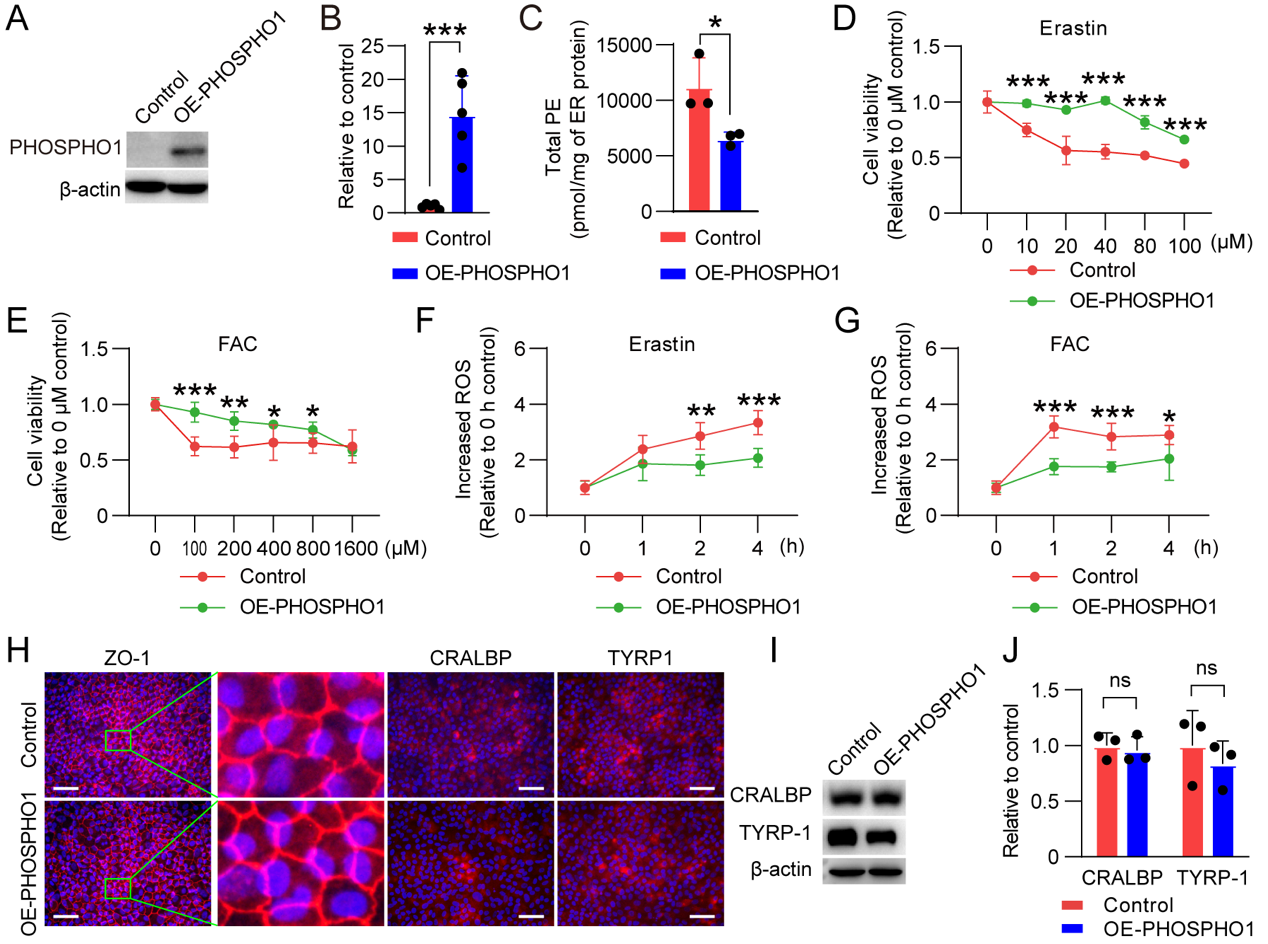
**

**Supplementary Fig. 7. PHOSPHO1 inhibits ferroptosis in iPSC-RPE cells.** (**A** and **B**) PHOSPHO1-variant 2 was overexpressed in iPSC-RPE cells and determined by (**A**) Western blotting and (**B**) quantitative analysis (n = 5). (**C**) The level of total PE molecular species was detected and determined as pmol/mg of ER protein (n = 3). (**D** and **E**) Cells were subjected to (**D**) 30 μM erastin or (**E**) 500 μM FAC treatment, and cell viability was analyzed by CCK-8 (n = 6). (**F**) and (**G**) Intracellular ROS was assessed using the DCFH-DA probe after 4 h of (**F**) erastin or (**G**) FAC treatment (n = 6). (**H**) Immunostaining of iPSC-RPE cells and PHOSPHO1-overexpressed iPSC-RPE cells. (**I** and **J**) The RPE specific markers CRALBP and TYRP-1 were determined by (**i**) Western blotting and (**j**) quantitative analysis (n = 3). Data are mean ± SD, *P < 0.05, **P < 0.01, ***P < 0.001 compared with control using unpaired two-sided t-tests. ns: no significance.

**Supplementary Fig. 8**

**
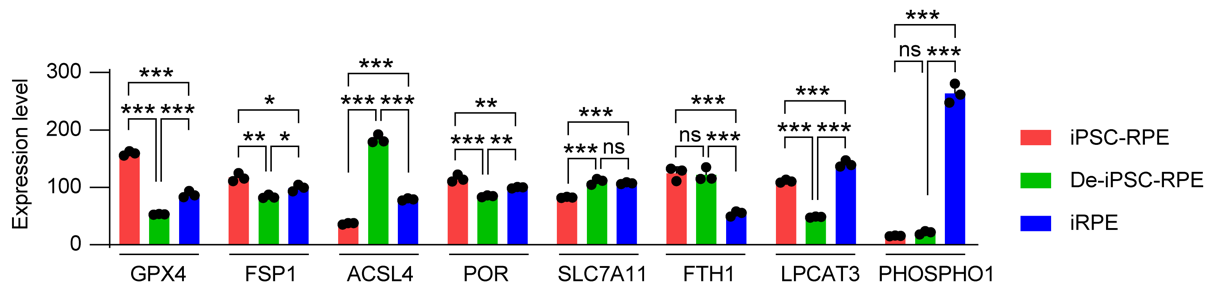
**

**Supplementary Fig. 8.** **The expression of ferroptosis-related proteins in iRPE, De-iPSC-RPE cells and iPSC-RPE cells.** Mass spectrometry was used to quantify the expressed ferroptosis-related proteins in iRPE cells, De-iPSC-RPE cells, and iPSC-RPE cells (n = 3). Data are mean ± SD, *P < 0.05, **P < 0.01, ***P < 0.001 using one-way ANOVA and post hoc Bonferroni’s test. The ns indicates no significance.

**Supplementary Fig. 9**


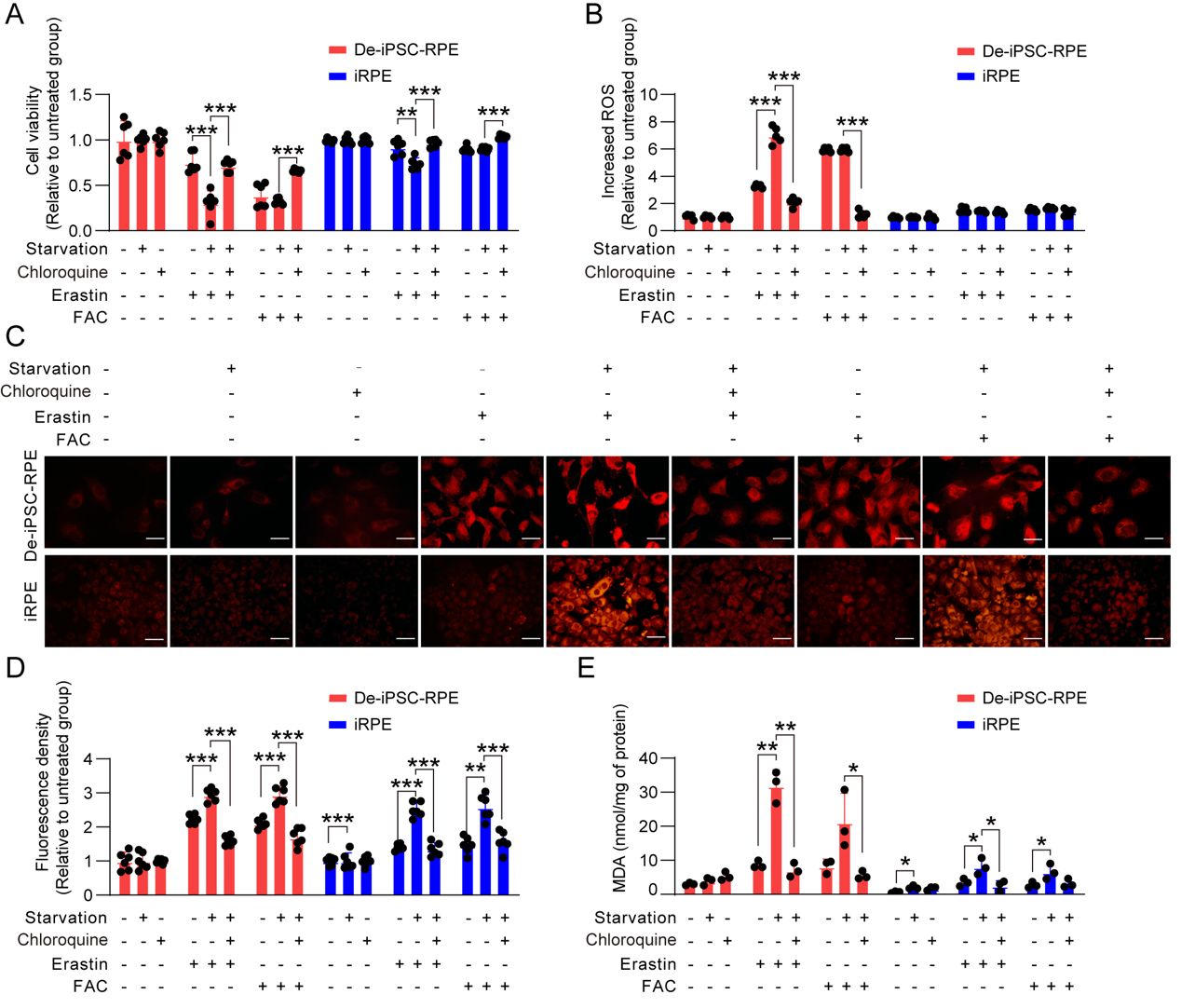


**Supplementary Fig. 9. Autophagy promotes ferroptosis.** De-iPSC-RPE cells and iRPE cells were cultured under starvation conditions to promote autophagy, with or without 100 μM chloroquine to inhibit autophagy, and then treated with 30 μM erastin or 500 μM FAC to trigger ferroptosis. (**A**) Cell viability was analyzed by CCK-8 kit (n = 6). (**B**) Intracellular ROS was measured with the DCFH-DA probe (n = 5). (**C**) Intracellular Fe^2+^ level was measured with the RhoNox-1 fluorescent probe and (**D**) quantified as fluorescence density relative to the untreated control (n = 6). (**E**) The MDA was measured by the MDA detection kit and determined as nmol/mg of protein (n = 3). Scale bar = 50 μm. Data are mean ± SD, *P < 0.05, **P < 0.01, ***P < 0.001 using one-way ANOVA and post hoc Bonferroni’s test.

**Supplementary Fig. 10**


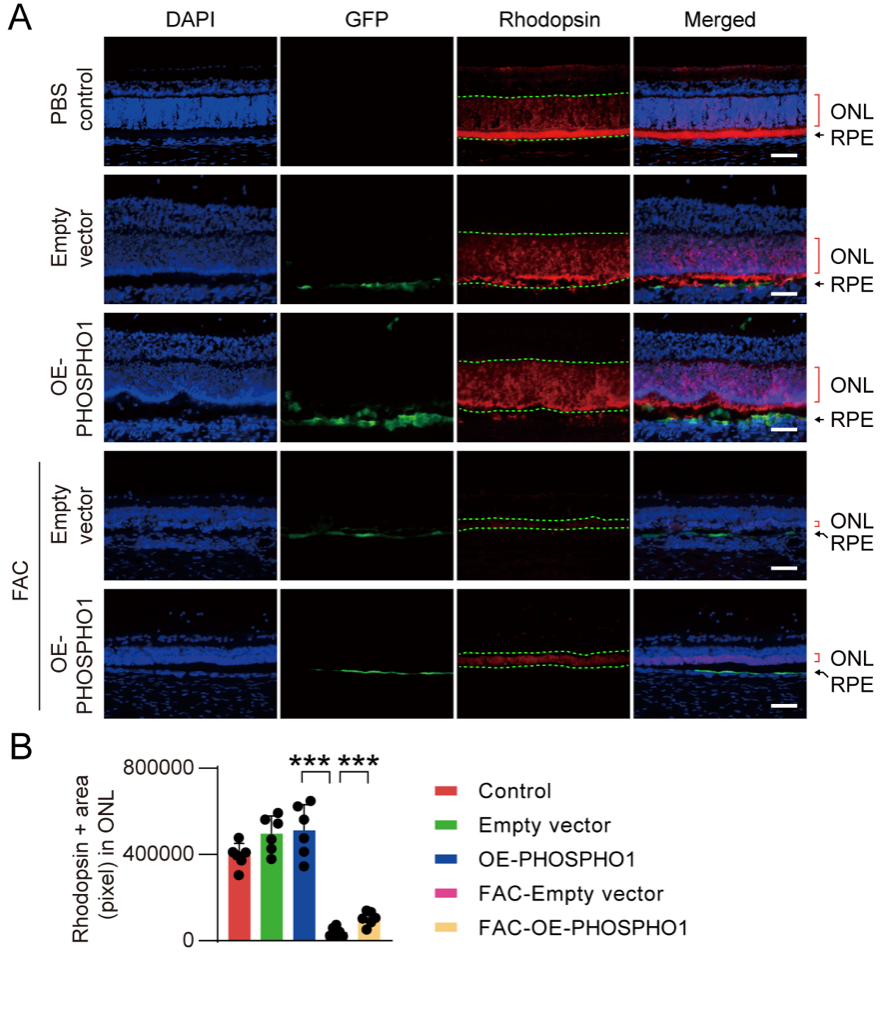


**
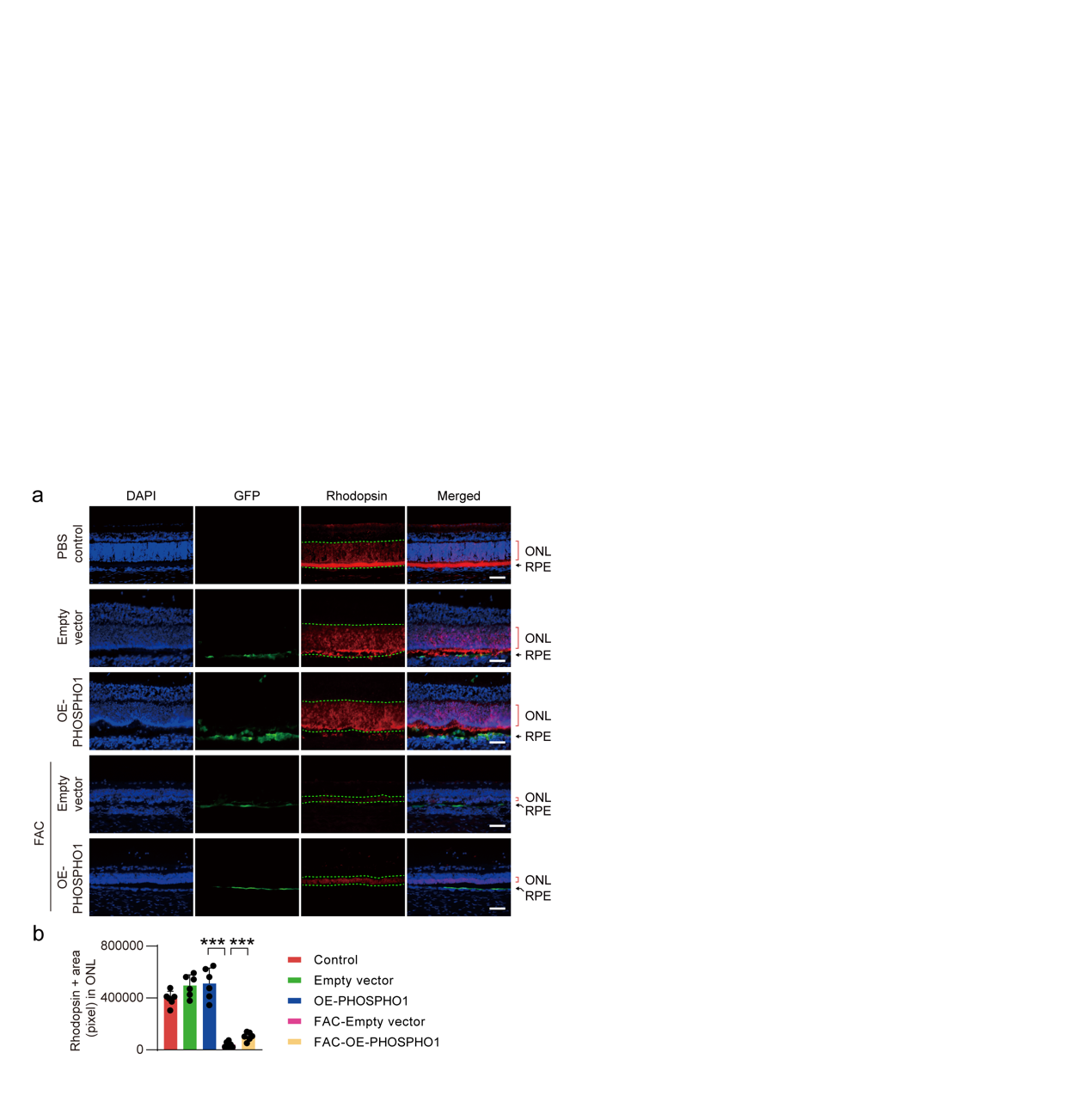
**

**Supplementary Fig. 10. Rhodopsin staining in retinal samples.** Cryosections of retina were subjected Rhodopsin staining. (**A**) Representative micrographs showing Rhodopsin immunostaining in retinal samples. (**B**) The expression level of Rhodopsin was determined as Rhodopsin+ area (pixel) in ONL (ONL was between green dashed lines) (n = 6). Scale bar = 50 μm. Data are mean ± SD, ***P < 0.001 using one-way ANOVA and post hoc Bonferroni’s test.

**Supplementary Fig. 11**


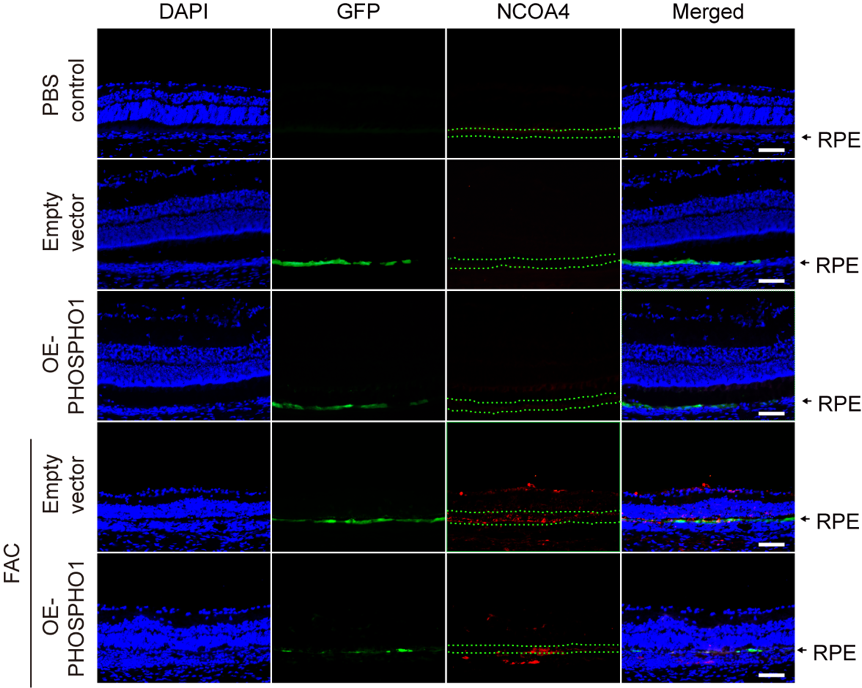


**Supplementary Fig. 11. NCOA4 staining in retinal pigment epithelial (RPE) cells.** Cryosections of retina were subjected to NCOA4 immunostaining. Representative micrographs showing NCOA4 immunostaining in RPE cells. Scale bar = 50 μm.

**Supplementary Fig. 12**

**
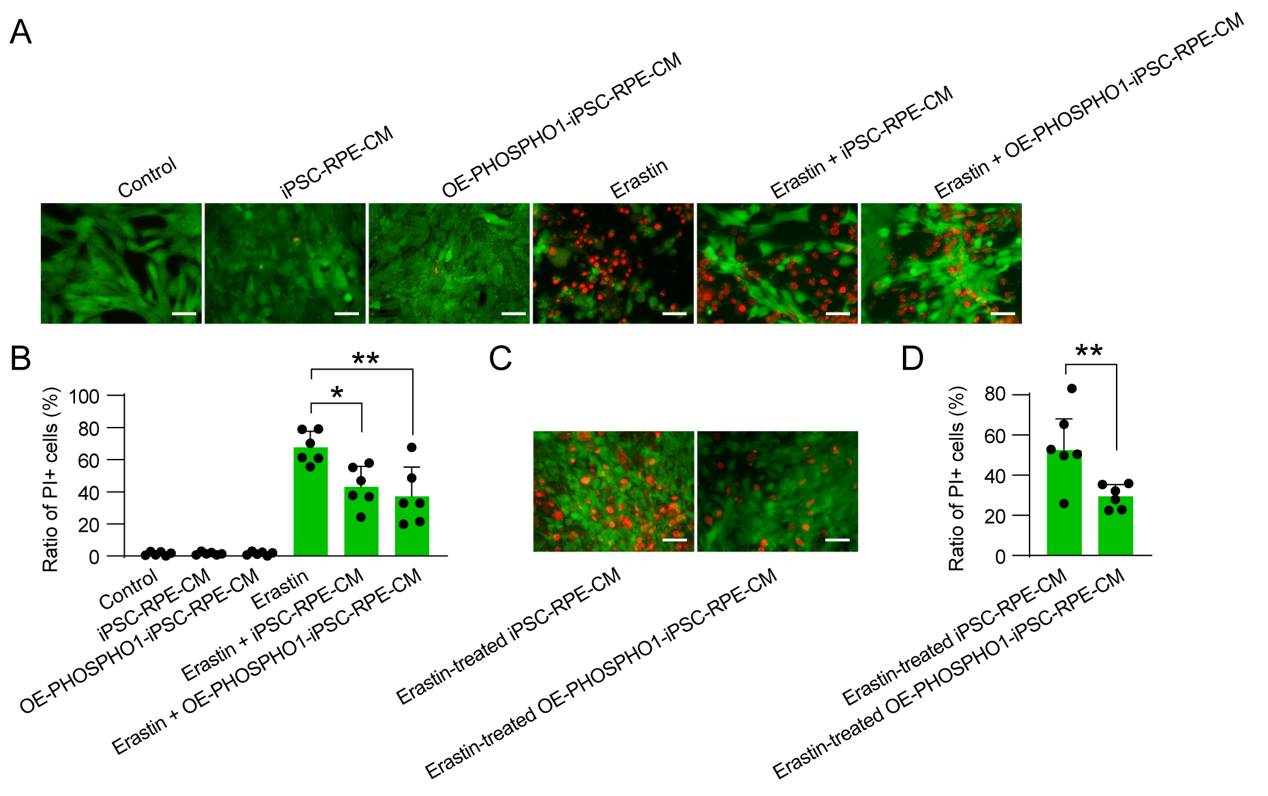
**

**Supplementary Fig. 12. 661W cell death is promoted by ferroptotic RPE cells.** (**A**) Calcein/PI staining of 661W cells treated with 1μM erastin after they were grown in iPSC-RPE-CM or OE-PHOSPHO1-iPSC-RPE-CM. (**B**) The ratio of PI+ cells was used to quantify the death of 661W cells (n = 6). (**C**) Calcein/PI staining of 661W cells treated with conditioned medium from erastin-treated iPSC-RPE cells or erastin-treated OE-PHOSPHO1-iPSC-RPE cells. (**D**) The ratio of PI+ cells was used to quantify the death of 661W cells (n = 6). Scale bar = 50 μm. Data are mean ± SD, *P < 0.05, **P < 0.01 using one-way ANOVA and post hoc Bonferroni’s test or Student’s t-test.

| Genes | Forward sequence (5’-3’) | Reverse sequence (5’-3’) | Accession no. |
| --- | --- | --- | --- |
| *PHOSPHO1-*variant1 | GGCCAACAGAGTCCCTACAG | GGCACATCCAACACCCTAGATA | NM_001386460.1 |
| *PHOSPHO1-*variant2 | ATCCTGCGCCCCAATACCT | CCATCCTGCCGTCCCTAGAT | NM_178500.4 |
| *PHOSPHO1*-total | ATCCGGGGGTGGATAAGACA | TTGTCGGTGCATTACCGTGA | NM_001386460.1 |
| *β-actin* | ACTCTTCCAGCCTTCCTT | GTACAGGTCTTTGCGGATG | NM_001101.5 |

**Supplemental Tables**

**Supplemental Table 1. Primers for qRT-PCR.**

**Supplemental Table 2. Antibodies for immunostaining and western blot.**

| Antibody | Company and Cat NO. | Dilution or final concentration | Application |
| --- | --- | --- | --- |
| ZO-1 | Proteintech, 21773-1-AP | 1:300 | immunofluorescence |
| FTL | Proteintech, 10727-1-AP | 1:1000 | western blot |
| NCOA4 | Absin, #abs100203 | 1:1000 | western blot |
| β-actin | Proteintech, 66009-1-Ig | 1:10000 | western blot |
| LC3I/II | Cell signaling technology,12741 | 1:1000 | western blot |
| PHOSPHO1 | SantaCruz Biotechnology; lot#10622 | 1:1000 | western blot |
| ZEB1 | Proteintech,21544-1-AP | 1:1000 | western blot |
| E-cadherin | Cell signaling technology,3195T | 1:1000 | western blot |
| ZO-1 | Proteintech, 21773-1-AP | 1:500 | immunofluorescence |
| CRALBP | Proteintech,15356-1-AP | 1:1000 | western blot |
| TRYP-1 | Abcam, ab235447 | 1:1000 | western blot |
| Rhodopsin | Abcam, ab98887 | 1:300 | immunofluorescence |
| 4-Hydroxynonenal | ThermoFisher, MA5-45792 | 1:300 | immunofluorescence |
| HRP-conjugated Affinipure Goat Anti-Rabbit IgG(H+L) | Proteintech, SA00001-2 | 1:3000 | western blot |
| HRP-conjugated Affinipure Goat Anti-Mouse IgG(H+L) | Proteintech, SA00001-1 | 1:3000 | western blot |
| Donkey anti-Rabbit IgG (H+L) Highly Cross-Adsorbed Secondary Antibody, Alexa Fluor™ 555 | Invitrogen, A31572 | 1:500 | immunofluorescence |
| Donkey anti-Mouse IgG (H+L) Highly Cross-Adsorbed Secondary Antibody, Alexa Fluor™ Plus 555 | Invitrogen, A32773 | 1:500 | immunofluorescence |

**Supplemental Table 3. The shRNA sequences for target genes.**

| shRNA | Sequence |
| --- | --- |
| sh*PHOSPHO1-*1 | TCCCTATCTATTCAGTT |
| sh*PHOSPHO1*-2 | GCGACCCCACACTATTT |
| sh*PHOSPHO1*-3 | CTACGAAGCCATCCCTT |
| sh*Control* | GCGCGATAGCGCTAATAATTT |
